# Ozone Reactions with Indoor Surfaces Soiled by Human Skin Oil are Impacted by Manner of Soiling, Relative Humidity, and Air Change Rate

**DOI:** 10.1021/acs.est.6c09797

**Published:** 2026-07-30

**Authors:** Wojciech Wojnowski, Armin Wisthaler, Tomas Mikoviny, Gabriel Bekö, Charles J. Weschler, Ann Sjöblom, Sarka Langer

**Affiliations:** † 49557 Gdańsk University of Technology, Faculty of Chemistry, Department of Analytical Chemistry, Gdańsk 80-233, Poland; ‡ 27255 University of Oslo, Department of Chemistry, Oslo N-0315, Norway; § International Centre for Indoor Environment and Energy, Department of Environmental and Resource Engineering, 12287 Technical University of Denmark, Lyngby DK-2800, Denmark; ∥ Environmental and Occupational Health Sciences Institute, Rutgers University, Piscataway, New Jersey 08854, United States; ⊥ IVL Swedish Environmental Research Institute, Environmental Chemistry, Göteborg SE-400 14, Sweden; # Chalmers University of Technology, Department of Architecture and Civil Engineering, Division Building Services Engineering, Göteborg 412 96, Sweden

**Keywords:** Skin oils, Squalene, Ozonolysis, PTR-ToF-MS, GC-MS

## Abstract

Human skin oil transferred to indoor surfaces is an important ozone sink and source of oxidation products, yet the effects of soiling mode and environmental conditions on this chemistry remain poorly understood. We examined ozone-initiated reactions on glass plates soiled by long-term natural accumulation, fresh squame deposition, or direct dermal contact at 20% or 70% relative humidity, air change rates of 6 or 12 h^–1^, and 42 ± 5 ppb ozone. Gas-phase products were quantified by PTR-ToF-MS, and surface residues by GC-MS. Ozone exposure consumed 22–98% of the squalene, depending on the soiling mode. Major gas-phase products included acetone, 4-oxopentanal (4OPA), and 6-methyl-5-hepten-2-one (6MHO); condensed-phase products included geranyl acetone (GA) and C_22_-aldehyde (4,9,13,17-tetramethyl-4,8,12,16-octadecatetraenal). Elevated relative humidity and lower air change rates enhanced ozone uptake and gas-phase product formation. Gas-phase product yields depended on relative humidity but not systematically on ventilation rate, whereas surface-bound product yields showed no systematic dependence on either parameter. Plates soiled by direct contact produced more than an order of magnitude higher volatile product mixing ratios than plates soiled with freshly deposited squames. These results show that soiling mode and environmental conditions strongly influence ozone-driven chemistry on skin-oil-contaminated indoor surfaces.

## Graphical abstract

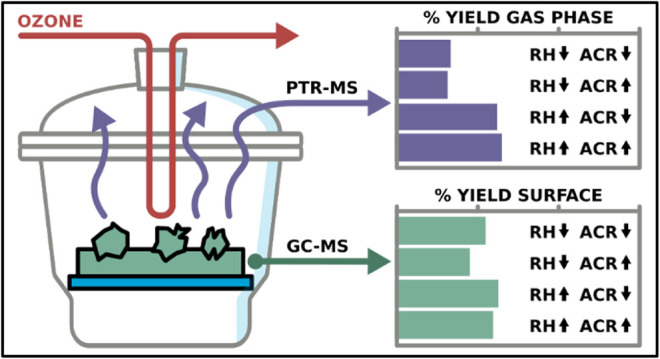

## 1 Introduction

Indoor ozone readily interacts with human skin oils on both occupant surfaces (skin, hair, clothing) and nonoccupant surfaces, the latter becoming soiled through desquamation, partitioning, and direct contact transfer. Approximately 15% of outdoor ozone entering a typical residence is consumed by reactions with skin oil, rising to as much as 55% in densely occupied spaces such as offices or classrooms.^1^ A meaningful fraction of these reactions occur on nonoccupant surfaces soiled with skin oil. Not only are skin-oil residues an important indoor ozone sink, but their reactions with ozone also generate volatile oxidation products that can contribute directly to occupant exposure via inhalation, as well as less volatile compounds that accumulate on surfaces.

Among the many human skin oil constituents, squalene (C_30_H_50_), an unsaturated triterpene containing six double bonds, is of particular interest because of its abundance and exceptionally high reactivity with ozone.^1–3^ It constitutes approximately half of the total unsaturation in skin oil. The ozone uptake by squalene-rich films is efficient, with a surface reaction probability substantially higher than for many other skin-oil lipids or common indoor surface films.^2,3^ The ozonolysis of squalene proceeds via multiple cleavage pathways of its double bonds, generating volatile, semivolatile, and relatively nonvolatile oxidation products.^3–5^ Key volatile carbonyl products include acetone, 6-methyl-5-hepten-2-one (6MHO), and 4-oxopentanal (4OPA), while semivolatile species include geranyl acetone (GA), longer-chain aldehydes, and organic acids that partition between surfaces and the gas-phase. Long-chain aldehydes, such as C_27_-pentaenal (4,9,13,17,21-pentamethyl-docosa-4,8,12,16,20-pentaenal), C_22_-tetraenal (4,9,13,17-tetramethyl-4,8,12,16-octadecatetraenal) and C_17_-trienal (5,9,13-trimethyltetradeca-4,8,12-trienal), as well as oligomeric products, remain on surfaces.^2,4^ Surface analyses have further identified oxidized residues, including carboxylic acids and higher-molecular-weight oligomers, which contribute to the persistence of chemically modified films on indoor surfaces.^2,4^

However, squalene is not the only ozone-reactive component of the lipid mix that constitutes human skin oil. A substantial fraction of skin-oil double bonds resides in unsaturated fatty acids and their associated glycerides and wax esters. Several of the most abundant unsaturated fatty acids, such as sapienic (cis-6-hexadecenoic) acid, which is notable for both its abundance and its specificity to human sebum,^6^ yield decanal as a major terminal product of ozonolysis.^1^ Taken together, these decanal precursors constitute approximately a third of all C=C bonds in skin-surface lipids, a contribution second only to that of squalene. Hence, decanal is an important and distinct marker of skin-oil-derived indoor ozone chemistry.^1^

Environmental conditions, together with the deposition pathway and the age of the skin oil film, strongly influence the yields and the phase distribution of skin oil ozonolysis products. Relative humidity (RH) has been shown to alter the mix of products: under dry conditions, the condensed phase tends to accumulate nonvolatile species such as organic acids and ozonides, whereas higher RH favors the formation and release of volatile carbonyls due to altered Criegee intermediate chemistry.^3,7^ Ventilation is another important factor, as lower air change rates promote thicker surface films and greater ozone uptake, while higher ventilation dilutes reaction products and limits their accumulation indoors.^7^ It was previously demonstrated that glass surfaces soiled for several weeks in a naturally ventilated residence accumulated nearly twice as much organic film mass as those in a mechanically ventilated residence, resulting in substantially higher ozone uptake.^8^ These aged films contained compounds characteristic of human skin oils, highlighting the contribution of occupant-derived material to indoor surface chemistry. Together, these findings indicate that both environmental conditions and the mode of soiling, whether via long-term accumulation, fresh squames deposition, or direct dermal contact, determine not only the rate of ozone consumption but also the spectrum of gas-phase and surface-bound products formed.

Most previous studies of ozone-skin oil chemistry have focused on either the gas-phase products or the oxidized residues remaining on surfaces, but rarely on both simultaneously. As a result, the connection between ozone uptake and product formation remains imperfectly understood. It is unclear how volatile byproducts that are inhaled by occupants relate to the less volatile compounds that accumulate on surfaces and can be ingested by occupants (e.g., hand-to-mouth activity). Moreover, while it is recognized that environmental factors such as RH and ventilation, as well as different modes of soiling, influence this chemistry, comparative studies that systematically assess the impact of these variables under controlled conditions are lacking. In this work, we investigate ozone-driven reactions on model surfaces that have been soiled in different ways and exposed to ozone under two relative humidities and two air change rates. Both gas-phase and surface products were quantified, allowing us to examine how environmental conditions and soiling mode jointly shape ozone uptake, product yields, and the balance between volatile and surface-bound products. The experimental conditions were selected to reflect realistic indoor environments, with ozone concentrations, relative humidity, and air change rates representative of those typically encountered indoors.

## 2 Materials and Methods

### 2.1 Study Design

Standard window glass sheets, 3 mm thick, were purchased from a local glazier and cut into square pieces, 18 × 18 cm, 324 cm^2^. The plates were washed with detergent in a laboratory dishwasher, rinsed with MiliQ-grade water, and dried in a heating cabinet for laboratory glassware. Afterward, the surfaces of the dry plates were rinsed and wiped with isopropanol-soaked sterile gauze pads and heated in an oven at 300 °C for 2 h. The plates were then mounted into 18 × 24 cm wooden photo frames.

Soiling of the plates by human-related indoor contaminants was achieved in four different ways. The plates were deployed in two occupied office rooms at IVL Swedish Environmental Research Institute in either horizontal or vertical orientation (i, ii) to collect human skin oils for a period of several months. Figures S1–S3 SI show the placement of the glass plates in the rooms, as well as the plates protected from soiling (blanks).

The horizontal plates were exposed to soiling for 111 days from October 6th, 2022, to January 25th, 2023. The vertical plates were exposed to soiling for 195 days from July 14th, 2022, to January 25th, 2023. After the soiling period, the photo frames holding the soiled glass plates were individually wrapped in aluminum foil and transported to the University of Oslo, Norway, for the indoor ozone chemistry experiments. Another set of plates was soiled in the horizontal orientation in an office (subject 1) and home environment (subject 2) for 1 week (iii). The amount of freshly shed squames in these samples was further enhanced by daily shaking the slept-in t-shirts of respective subjects over the plates during the week-long soiling, prior to the controlled exposure to ozone. Yet another set of plates (iv) was soiled with skin oils by simulated direct dermal contact (rubbing) of the plates against an overnight slept-in cotton t-shirt of a third subject.

Indoor air temperature and RH were continuously monitored during the preparation of the horizontally and vertically soiled plates using a Wöhler CDL210 sensor/logger (Wöhler Technik GmbH, Germany; calibrated according to the user’s manual) with a time resolution of 2 min. Using diffusive ozone samplers^9^ located outdoors and within the two offices, an indoor-to-outdoor ozone ratio of 0.129 ± 0.004 was measured during three one-month periods. The long-term average ozone concentration in the office rooms was estimated to be 6–10 μg m^–3^ (3–5 ppb) from the established indoor-to-outdoor ratio and outdoor ozone concentration for the soiling period measured at a nearby urban ambient air quality monitoring station. The average temperature and RH during the soiling period were 21 ± 0.5 °C and 37 ± 12%, respectively (see Table S1, SI for details). Indoor air temperature and RH were not measured during the fresh (iii) and dermal contact (iv) soiling; they were assumed to be at typical indoor levels.

The soiled plates were exposed to ozone in the chamber described below. The duration of the exposure was up to 140 min and depended on the approximate time at which the target gaseous reaction products were either exhausted or reached steady state. Gaseous products were monitored using Proton Transfer Reaction Time-of-Flight Mass Spectrometry (PTR-ToF-MS). Surface-bound reaction products remaining on the glass plates were collected by wiping the surfaces twice with isopropanol-soaked, precleaned sterile gauze pads. The pads were wrapped in aluminum foil, stored in tightly capped glass vials in a freezer (−20 °C) prior to analysis, and analyzed using Gas Chromatography–Mass Spectrometry (GC-MS). The same procedure was used to determine the pre-exposure loadings on plate replicates and on plates used as blanks (not soiled).

### 2.2 Experimental Setup

A schematic representation of the experimental setup is shown in Figure 1. Its central element was a glass desiccator used as the reaction chamber (Figure S4, SI). Its internal volume with the lid on was 20.01 dm^3^ without the armature and sample (i.e., the inlet, perforated ceramic base plate, glass plate, and plastic spacers) and 19.50 dm^3^ with the armature and sample. The internal surface area of the chamber was 0.389 m^2^ and 0.638 m^2^ without and with the armature, respectively.

**Figure 1:**
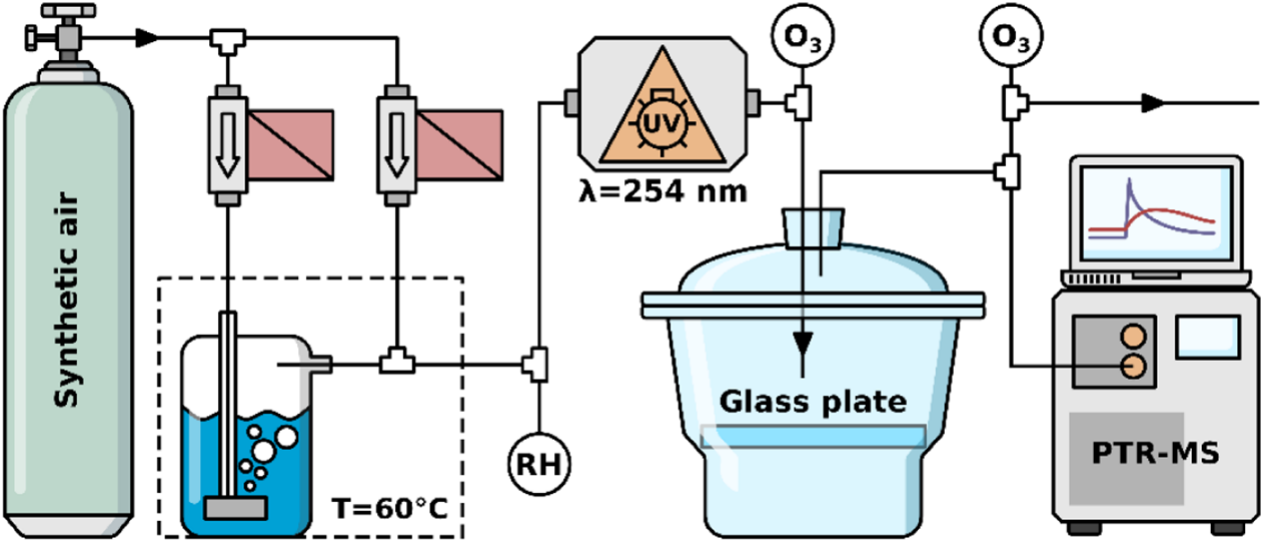
A schematic representation of the experimental setup.

Synthetic air (Nippon Gases, Oslo, Norway) flow was controlled using a pair of mass flow controllers and directed through a 1/4″ perfluoroalkoxy alkane (PFA) line. Part of the flow was passed through a bubbler incubated at 60 °C to obtain the desired RH. Ozone was generated using an in-line ozone generator equipped with a Pen-Ray Hg 254 nm UV light source (UVP Ltd., Cambridge, UK). Ozone concentration was monitored upstream and downstream of the desiccator using the Thermo 49C (Thermo Fisher Scientific, Waltham, MA, USA) and the 2B Tech Model 205 (2B Technologies, Broomfield, CO, USA) O_3_ monitors, respectively. The upstream monitor was used to set the inlet ozone concentration to its targeted value of ∼40 ppb before it entered the chamber (mean of 42 ± 5 ppb). The downstream monitor measured ozone concentrations in the chamber (i) prior to (i.e., a time-weighted average from the steady-state measurement 5 min before t_0_) and (ii) during reaction with the glass plates. Although (i) would more rigorously be described as the “empty chamber” concentration, it is denoted as “*noplate*” elsewhere in the manuscript for simplicity. The difference between these “*noplate*” and “*plate*” chamber concentrations (ΔO_3_) measured downstream was used to calculate the amount of ozone removed by the glass plate (Figures 2 and 3). A small fraction of the downstream flow was subsampled via a 1/16″ PFA line into the PTR-ToF-MS analyzer.

**Figure 2:**
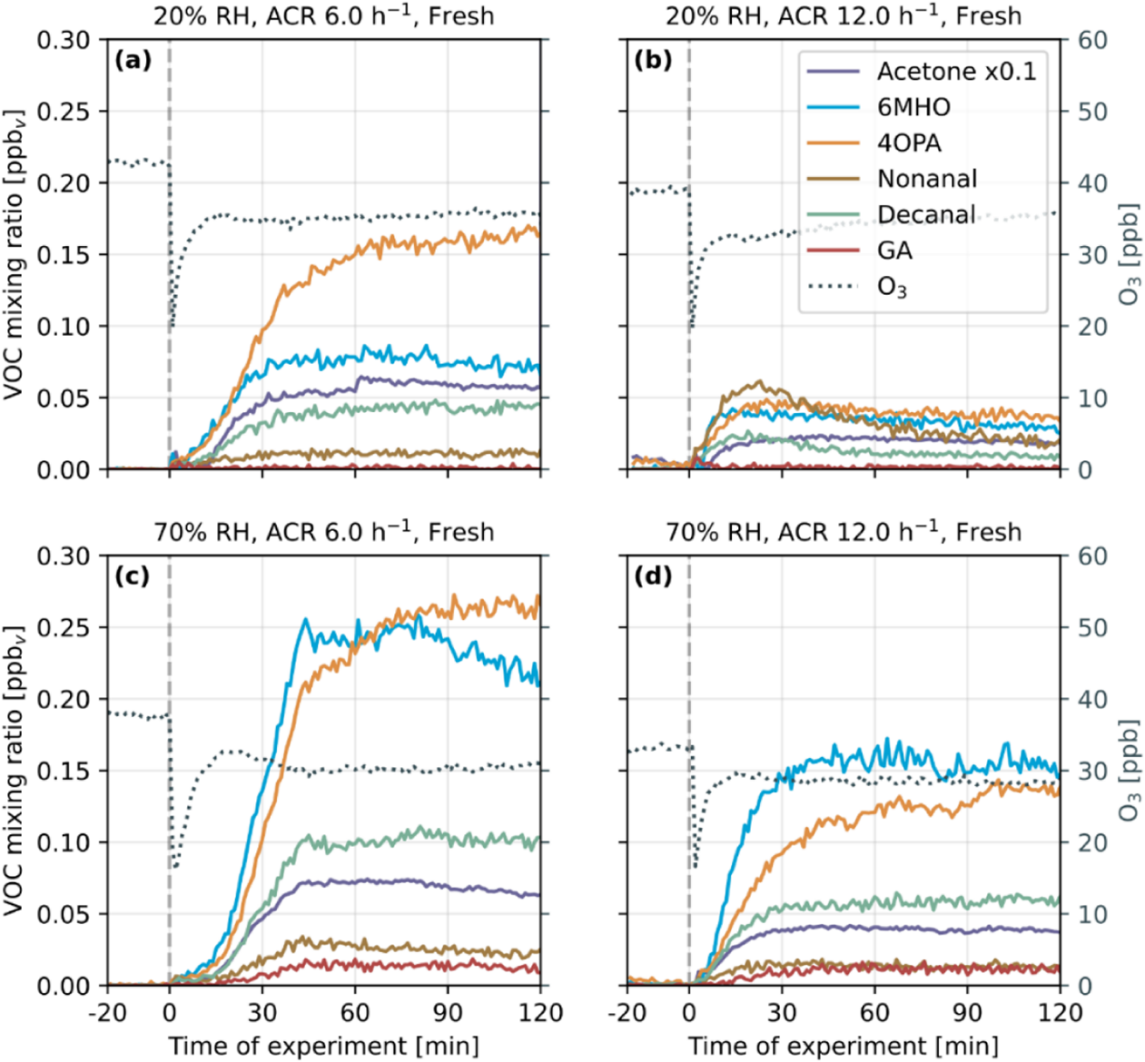
Corrected mixing ratios of the monitored compounds for the duration of the experiments under different measurement conditions in the “Fresh” scenario and the corresponding O_3_ mixing ratios. Note the scaling of the acetone mixing ratios by 0.1 for clarity.

**Figure 3:**
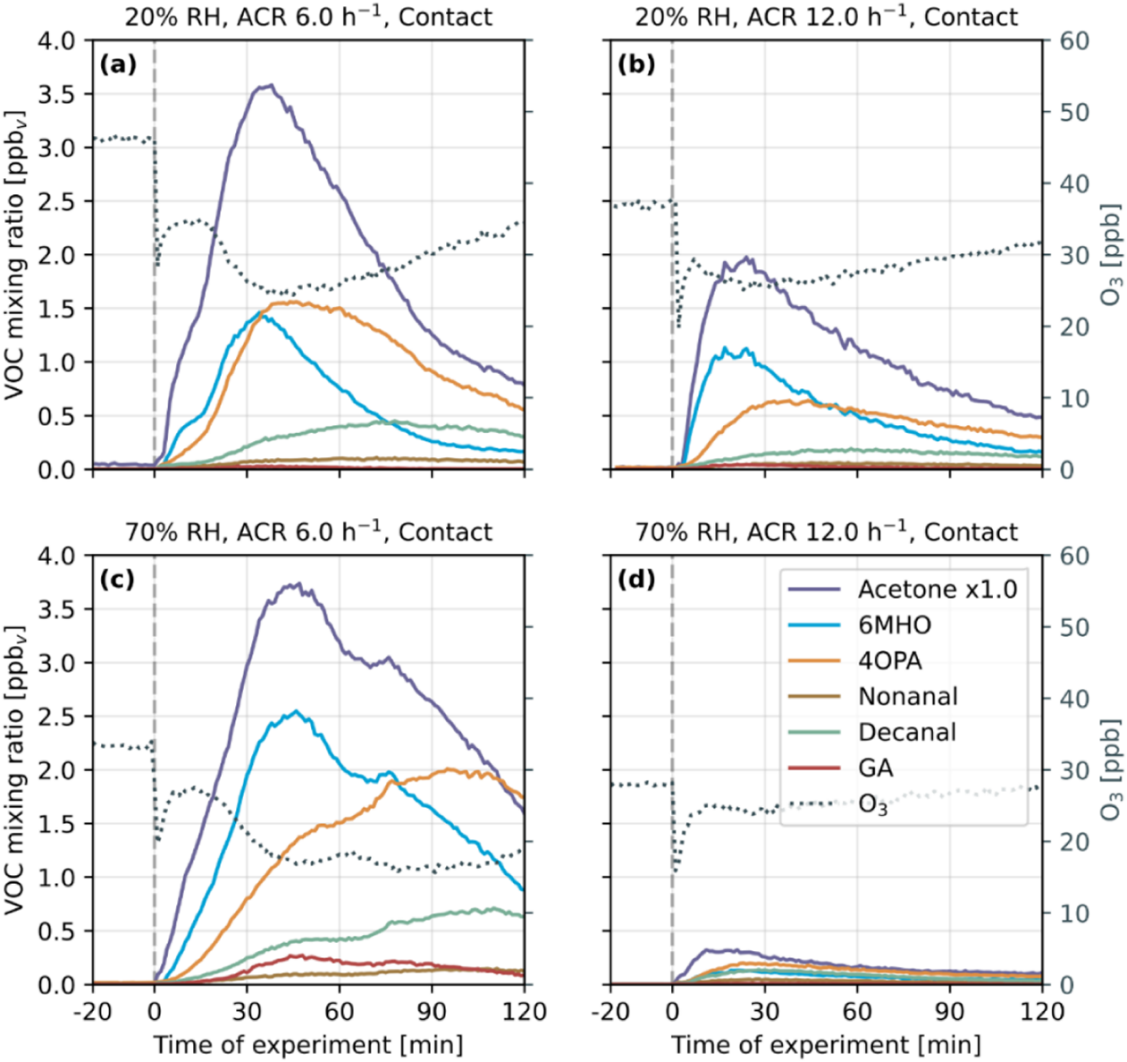
Corrected mixing ratios of the monitored compounds for the duration of the experiments under different measurement conditions in the “Dermal contact” scenario and the corresponding O_3_ mixing ratios. The relatively low mixing ratios in subplot (d) are almost certainly the result of an experimental error.

The experimental matrix consisted of a single (room) temperature of approximately 23 °C, relative humidity of 20% and 70%, and air flow rates through the reaction chamber of 2 dm^3^ min^–1^ and 4 dm^3^ min^–1^, resulting in air change rates (ACR) of 6 h^–1^ and 12 h^–1^, respectively. Table 1 summarizes the experimental setup for the ozone chemistry experiments in the chamber.

**Table 1:** Overview of the Type of Plate Soiling and Conditions of RH and ACR in the Chamber Experiments

Type of soiling	Soiling time
Horizontal	16 weeks
Vertical	28 weeks
Fresh	1 week
Dermal contact	Immediate

matrix for ozone exposure experiments for all types of soiling Soiling time	
RH 20%	ACR 6 h^–1^
	ACR 12 h^–1^
RH 70%	ACR 6 h^–1^
	ACR 12 h^–1^

Clean glass plates, wrapped in aluminum foil immediately after washing and heating, were used as blanks for the soiled plates. These clean plates were subsequently unwrapped just before being exposed to ozone in the chamber, and their surfaces were later wiped in the same manner as the soiled plates exposed to ozone. The values of Δozone for the blank experiments, at the appropriate combinations of RH and ACR, represent ozone loss on the clean glass plates. Loss to a clean plate was subtracted from the corresponding value for the experiment with a soiled plate to obtain the amount of ozone lost solely to the reaction with the soiled surface, ΔO_3_,_reacted_ (eq 1). These blank corrected values were then used in further determination of both gas-phase and surface-phase yields of the reaction products.

1 $$\Delta O_{3,RH,ACR,reacted}=\Delta O_{3,RH,ACR,sample}-\Delta O_{3,RH,ACR,blank}$$

Two experiments were performed in duplicate: plates soiled by dermal contact at 20% RH and ACR of 12 h^–1^, and freshly soiled plates at 20% RH and 12 h^–1^. Surface wipes were also collected from glass plates soiled via each of the above-mentioned conditions but not exposed to ozone.

### 2.3 Chemical Analyses

The target gaseous analytes monitored using PTR-ToF-MS and analyzed in the wipe samples were the most abundant products from ozonolysis of squalene and unsaturated fatty acids and triglycerides present in skin oil. These were the primary squalene products: acetone, 6MHO, and geranyl acetone (GA), the secondary squalene product 4OPA, and primary products from oleic acid and sapienic acid (and their related triglycerides): nonanal and decanal.^5^

The PTR-ToF-MS analyzer was custom-built and described in detail elsewhere.^10^ It can measure most VOCs in real-time with high mass resolution. The instrument was operated in the H_3_O^+^ mode, with the reaction chamber set to 2.75 mbar, 120 °C, and 550 V. It was calibrated using a certified gas calibration mixture (Apel-Riemer Environmental, USA) at different RHs. The target analytes were calibrated for using aqueous standard solutions (Sigma-Aldrich, Norway) introduced via a liquid calibration unit (Ionicon Analytik, Innsbruck, Austria), except for acetone, which was calibrated based on the gas standard, and geranyl acetone, where the sensitivity to acetone was used instead. The obtained mass spectra were integrated every 5 s. Data were preprocessed using the PTR-MS Viewer v. 3.4 software (Ionicon Analytik, Innsbruck, Austria). Target analytes were assigned to particular peaks based on the exact mass of the parent ion.

The surface-bound reaction products were extracted from the wipes using a modified procedure described previously.^11^ For the chemical analysis, the wipe samples were spiked with isotopically labeled DEHP-d_4_ internal standard, extracted and analyzed using GC-MS in the Total Ion Chromatogram (TIC) mode. Five-point calibration curves were used to quantify squalene, 6MHO, geranyl acetone, nonanal, decanal, and pentadecanoic acid. Details of the procedures for the chemical analyses, detection limits based on the analyses of procedural blanks (clean sterile gauze pads), and the analysis of replicates (multiple injections of selected samples) are provided in the SI (Supporting Text S1 and Table S2). Details of the measurement reproducibility, averages and standard deviations from analyses of the duplicate samples are provided in Table S3. Based on duplicate measurements that were conducted for two experiments, the relative standard deviations for the targeted compounds were in the range of 0.30–17%. The propagated relative uncertainties (Table S3) were estimated from duplicate-yield repeatability, compound-specific calibration/quantification uncertainty, and ΔO_3_ precision, and ranged from 6 to 30% for O_3_-normalized gas-phase yields, 10–30% for O_3_-normalized surface-bound yields, 10–30% for squalene-normalized gas-phase yields, and 10–30% for squalene-normalized surface-bound yields for particular analytes. The uncertainties for the overall yields in the different scenarios ranged from 5% to 20%.

Unfortunately, we were unable to determine the exact removal efficiency of wiping the materials off the glass surfaces. An addition of a unique standard prior to wiping would have helped to assess the completeness of removal of the material deposited on the surfaces. However, the quantity used in further analyses is the difference between the initial and the final amount of squalene on the surfaces, ΔSqualene. We did maintain a reproducible wiping procedure for the glass surfaces that were soiled in different ways.

The amounts of squalene and surface-bound reaction products were converted from μg/plate to nanomol/plate through the molecular weight of the substances. The concentrations of gaseous compounds, ozone, and the reaction products were converted from ppb to μg m^–3^ at standard temperature and pressure. The amounts of ozone and products in nanomoles per experiment were scaled to the actual volume of air that passed through the chamber during the experiment, calculated from the air flow through the chamber (either 2 L min^–1^ or 4 L min^–1^) and the duration of each experiment (Table 3).

## 3 Results and Discussion

### 3.1 Ozone and Squalene

Ozone deposition velocities (v_d_) and removal rates from plates (*k*_p_), corrected for background chamber losses, were calculated for all soiling conditions (see Supporting Text S2 for details and Table S4 for ozone mixing ratios). The steady-state v_d_ values for the horizontally (0.003 ± 0.001 cm s^–1^) and vertically (−0.002 ± 0.005 cm s^–1^) soiled glass plates (uncorrected) did not differ significantly from the blanks (0.005 ± 0.002 cm s^–1^), which is consistent with the weak reactivity of aged surface films reported previously.^8^ The steady state v_d_ values for the blanks and the vertically and horizontally soiled surfaces are the average ± standard deviation of the individual values across the four RH and ACR combinations. In contrast, freshly soiled plates exhibited v_d_ values between 0.017 cm s^–1^ (20% RH, 6 h^–1^) and 0.025 cm s^–1^ (20% RH, 12 h^–1^), at the lower end of values reported for indoor materials such as wooden panels or paints.^12,13^ In the case of the plates soiled through direct dermal contact, the calculated v_d_ did not reach steady state during the >120 min of the experiment. However, transient v_d_(t) maxima of approximately 0.1 cm s^–1^ closely matched values observed for textiles soiled with skin oil (0.15 ± 0.02 cm s^–1^).^14^ These results indicate that while thin, vertically and horizontally deposited films behave more like weakly reactive background surfaces, thicker transfer films from direct skin contact or intentionally added skin flakes can approach the uptake efficiency of soiled textiles under similar chamber conditions. This is consistent with earlier reports of high reactive uptake on squalene-containing films^2,3^ and reinforces the conclusion that skin oils transferred by contact to surfaces play a disproportionate role in indoor ozone removal.^1^ Still, the ozone deposition velocity directly to human surfaces, v_d‑h_, reflecting the combined contribution of skin, hair, and clothing, can be considerably higher: ∼0.4 cm s^–1^.^1,5,15^

The rate at which ozone was removed by the clean (blanks) and the skin oil-soiled surfaces in the chamber (i.e., desiccator) was calculated for additional characterization of ozone behavior in the experiments (Supporting Text S2, Table 2). The ozone removal rate coefficients for the blanks in the reaction chamber (0.29–0.68 h^–1^) were of the same order of magnitude as those determined for an empty stainless-steel chamber of 0.15 h^–1^ and 0.17 h^–1^ (range 0.092–0.29 h^–1^).^16,17^ The “blank” *k*-values describe the ozone being decomposed on the surfaces of the clean plates and all other internal surfaces in the chamber. The slightly higher *k*-values from our blank experiments, compared to the *k*-values from the stainless-steel chamber, likely reflect the different smoothness of the materials. The *k*-values of the freshly soiled surfaces resemble the corresponding values from experiments using soiled (slept-in overnight) t-shirts (1.22 h^–1^) as the source of skin oils, in a chamber ozonolysis experiment.^16^ The values of removal rate coefficients of ozone reacting with skin oils on soiled occupant and nonoccupant surfaces of 3.1 h^–1^–9.4 h^–1^ are similar to the values from surfaces soiled by dermal contact.^1^ With respect to removal rate coefficients and deposition velocities, ozone in our experiment behaves as expected for clean surfaces and surfaces soiled by skin oil.

**Table 2:** Ozone Removal-Rate Coefficients (*k*) and Deposition Velocities (v_d_) to Plates Calculated Using eqs S1–S5 in the Supporting Text S2

Soiling condition	Scenario	k [h^–1^]	v_d_/v_d_(t)_max_ [cm s^–1^]
Blank	20% 6 h^–1^	0.29	
Blank	20% 12 h^–1^	0.42	
Blank	70% 6 h^–1^	0.32	
Blank	70% 12 h^–1^	0.68	
Fresh	20% 6 h^–1^	1.10	0.017
Fresh	20% 12 h^–1^	1.56	0.019
Fresh	70% 6 h^–1^	1.16	0.025
Fresh	70% 12 h^–1^	1.39	0.024
Dermal cont.	20% 6 h^–1^	2.75	^a^0.086
Dermal cont.	20% 12 h^–1^	4.00	0.102
Dermal cont.	70% 6 h^–1^	4.00	0.107
^b^Dermal cont.	70% 12 h^–1^	0.48	0.024

a In the case of the dermal contact experiments, the v_d_ values are the maximum transient velocities in lieu of values for the steady state, which was not reached in that scenario.

b Experimental error.

Surface loadings of squalene on the plates prior to ozone exposure could not be determined directly on the same plates used in the chamber experiments. Since the deposited skin oils were unlikely to be distributed uniformly across each plate, and because the total amount transferred could not be assumed to be identical between plates, removing a portion of the film before exposure risked leaving too little material for the ozonolysis experiments. Therefore, for each soiling scenario, one representative soiled plate was wiped immediately before the remaining plates were placed in the chamber, while the chamber-exposed plates were wiped after ozone exposure. All wipes were analyzed for squalene and pentadecanoic acid (C_15_-monocarboxylic acid). Pentadecanoic acid was used as a reference compound because it is a unique constituent of skin oil and, as a saturated fatty acid without C=C bonds, is relatively unreactive with ozone, whereas squalene is highly ozone-reactive.^18–21^ Hence, the ratio of squalene to pentadecanoic acid is informative with respect to the amount of squalene on a soiled plate immediately before a plate was placed in the chamber and exposed to ozone. The amount of pentadecanoic acid measured on a plate postexposure was used to estimate the amount of squalene initially present on each plate immediately before it was exposed to ozone in the chamber. This is how the values for “Squalene initial” in Table 3 were obtained. The postexposure amount of squalene on a plate was measured directly (“Squalene final” in Table 3).

**Table 3:** Squalene and Ozone from All Experiments^a^

Soiling condition	Scenario	Exp. dur.	Squalene initial	Squalene final	Δ*S*qualene	% Squalene reacted	O_3 noplate_	O_3 plate_	ΔO_3_	%O_3_ reacted
Blank	20% 6 h^–1^	59	n.d.	0.32	n.a.	n.a.	232	220	12	5.4
Blank	20% 12 h^–1^	78	n.d.	0.50	n.a.	n.a.	501	484	17	3.4
Blank	70% 6 h^–1^	51	n.d.	0.27	n.a.	n.a.	137	130	7	5.1
Blank	70% 12 h^–1^	90	n.d.	0.25	n.a.	n.a.	456	432	24	5.3
Vertical	no ozone	n.a.	0.78	n.a.	n.a.	n.a.	n.a.	n.a.	n.a.	n.a.
Vertical	20% 6 h^–1^	56	0.78	0.29	0.49	63%	223	215	8	3.4
Vertical	20% 12 h^–1^	56	0.78	0.24	0.54	69%	357	346	11	3.0
Vertical	70% 6 h^–1^	47	0.78	0.18	0.60	77%	149	137	12	8.1
Vertical	70% 12 h^–1^	67	0.78	0.24	0.54	70%	342	336	6	1.9
Horizontal	no ozone	n.a.	3.3	n.a.	n.a.	n.a.	n.a.	n.a.	n.a.	n.a.
Horizontal	20% 6 h^–1^	61	3.7	1.7	2.0	54%	240	222	18	7.6
Horizontal	20% 12 h^–1^	30	3.1	1.9	1.3	40%	188	172	16	8.4
Horizontal	70% 6 h^–1^	39	3.1	2.0	1.1	36%	112	100	12	10.5
Horizontal	70% 12 h^–1^	34	2.5	2.0	0.5	22%	176	169	7	3.9
Fresh	no ozone	n.a.	61.5	n.a.	n.a.	n.a.	n.a.	n.a.	n.a.	n.a.
Fresh	20% 6 h^–1^	121	58.0	20.3	37.7	65%	422	343	79	18.8
Fresh	20% 12 h^–1^	126	112.2	78.9	33.3	30%	801	689	113	14.1
Fresh	70% 6 h^–1^	129	58.1	17.9	40.2	69%	611	490	121	19.8
Fresh	70% 12 h^–1^	132	69.1	41.1	28.0	39%	717	612	105	14.7
Dermal cont.	no ozone	n.a.	25.3	n.a.	n.a.	n.a.	n.a.	n.a.	n.a.	n.a.
Dermal cont.	20% 6 h^–1^	141	30.0	0.6	29.4	98%	531	353	179	33.6
Dermal cont.	20% 12 h^–1^	135	31.3	0.9	30.4	97%	786	575	211	26.8
Dermal cont.	70% 6 h^–1^	140	32.3	1.5	30.8	95%	381	221	160	41.9
Dermal cont.	70% 12 h^–1^	119	34.0	1.9	32.1	94%	544	496	48	8.8

a Squalene is given in nmol/plate, ozone is given in nmol/exposure, and the experiment duration is given in minutes. The LOD of squalene was 0.08 nanomol/plate; N.A. = not analyzed; N.D. = not detected. Note that the gas-phase measurements for the dermal contact, 70% 12 h, ozone is given in nanomol/exposure, and the experiment duration is in minutes. The LOD of squalene was 0.08 nanomol/plate; N.A. = not analyzed; N.D. = not detected. Note that the gas-phase measurements for the dermal contact, 70% 12 h^–1^ experiment were likely affected by an experimental error.

The initial and final amounts of squalene on the surfaces and of ozone in the gas phase for each soiling, airflow, and humidity scenario are summarized in Table 3. Blank-corrected ΔO_3,reacted_ values were used in further determination of both gas-phase and surface-phase yields of the reaction products. The correction for blank experiments may seemingly result in negative values of ΔO_3_ for the vertically and horizontally soiled surfaces. However, the ΔO_3_ for the blanks and for the vertically and horizontally soiled surfaces were not significantly different (two-tailed *t* test, p = 0.1773 for the vertically soiled surfaces; p = 0.7024 for the horizontally soiled surfaces).

Markedly different amounts of skin oils were retained on the surfaces soiled through different processes. In the case of the vertically soiled plates, the concentration of pentadecanoic acid was below the LOD, and thus the initial concentration of squalene could not be determined for these plates (n.d. in Table 3). Hence, for vertically soiled plates (i) we assumed the initial concentrations of squalene to be the same as on the unexposed surface, i.e., 0.78 nanomol/plate (Table 3). On the surfaces soiled in the horizontal position (ii), the initial amounts of squalene were approximately 2.5–3.7 nanomol/plate; on the freshly soiled surfaces (iii), 58–112 nanomol/plate; and on surfaces soiled by dermal contact (iv), approximately 30 nanomol/plate. This translates on average to 1.0 ng cm^–2^, 4.0 ng cm^–2^, 91 ng cm^–2^, and 39 ng cm^–2^ for the plates soiled vertically, horizontally, freshly, and through dermal contact, respectively. For reference, we have found the average concentration of squalene on human skin surface to be 2140 ng cm^–2^ (54–6320 ng cm^–2^ range) in our previous work.^11^ The initial amounts of squalene differed significantly between the different soiling scenarios (one-tailed *t* test; p from <0.00001 to 0.035), with one exception: its initial amount on the surfaces soiled freshly was not significantly different from that on the surfaces soiled through dermal contact (p = 0.169). These values, while one to 2 orders of magnitude lower than typical skin surface loadings, are still capable of sustaining appreciable ozone uptake.^8^

The initial amount of squalene on a soiled plate in units of ng/cm^2^ was converted to an estimated contribution to overall film thickness using a density of 0.86 g/cm^3^ for squalene. The calculated contribution was 0.01, 0.05, 1.1, and 0.45 nm for vertically, horizontally, freshly, and dermally soiled plates, respectively. This estimated contribution of squalene to overall film thickness was lowest for vertically soiled plates, consistent with the slow deposition of skin-oil-containing squames on vertical indoor surfaces. The comparable pentadecanoic acid amounts measured on horizontally soiled surfaces and dermal-contact plates indicate that similar amounts of skin oil had been deposited in these two scenarios. However, on the horizontal plates, co-occurring squalene was continuously deposited and simultaneously reacted with the low levels of ozone present during the months-long soiling period, whereas pentadecanoic acid, which contains no C=C bonds, is expected to have reacted much more slowly.

The amount of squalene that reacted was approximately 70% on the vertically soiled plates and 22–54% on the horizontally soiled plates, consistent with the influence of film aging and morphology on ozone accessibility observed previously.^7^ On freshly soiled (one-week) surfaces, the proportion of squalene reacted was nearly 70% at an ACR of 6 h^–1^ and ∼35% at an ACR of 12 h^–1^, at both relative humidities. Similarly, on the horizontally soiled surfaces, the proportion of squalene reacted was larger at 6 h^–1^ than at 12 h^–1^ at identical RH. On these surfaces, the proportion of squalene reacted was much larger at lower RH than at higher RH at identical ACR. This was not the case for other soiling conditions. Nearly all squalene from the dermal-contact plates reacted with ozone (94–98%), in line with studies showing near-quantitative consumption of squalene via ozonolysis in similar scenarios.^4^ For the vertically soiled plates, the proportion of squalene that reacted was approximately the same across RH and ACR scenarios, although the fact that pentadecanoic acid was beneath its LOD on vertically soiled plates impeded our ability to accurately estimate the initial amount of squalene on the vertical plates.

### 3.2 Reaction Products in the Gas Phase

The initial spikes of acetone and 6MHO, visible in the volume mixing ratio profiles of the PTR-ToF-MS measurements (SI, Figures S5–S9), were caused by the introduction of ambient laboratory air when the chamber was briefly opened to insert the samples. These signals appeared consistently in the blank experiments (SI, Figure S9). Opening the chamber also produced a short-term decrease in ozone concentration, which returned to steady state within ∼15 min. The interferences from acetone and 6MHO were corrected as described in Supporting Text S3 and illustrated in Figure S10 (SI). Time-weighted averages (TWAs, ppb_V_) were calculated from the corrected tracers and converted to μg m^–3^ at 25 °C and 1013 hPa. The total air volume sampled was derived from the flow rates of 2 and 4 dm^3^ min^–1^ and the duration of the experiment. The resulting gaseous product amounts are reported as nanomoles in Table S5.

The concentrations vs time profiles of the target gaseous products of squalene ozonolysis from the PTR-ToF-MS measurements, corrected for the interference from ambient acetone and 6MHO, are shown in Figure 2 for the freshly soiled plates and in Figure 3 for the plates soiled with the human skin oils by simulated direct dermal contact. The subplots are truncated at 120 min for clarity, and actual durations of particular experiments are given in Table 3. The results shown in Figure 3d are indicative of an error (unidentified) in the experiment—after subtracting the laboratory air interference from the “Vertical” and “Horizontal” samples, the remaining signals resembled random noise below the LOD. For reference, their uncorrected versions are shown in Figures S5 and S6 in the SI. Figure S12 shows mixing ratios of the ozone reaction products from a duplicate experiment at 20% RH and ACR of 12 h^–1^ with the freshly soiled surface.

For the plates soiled both “freshly” and through dermal contact, the integrated gas-phase product amounts were generally higher at 6 h^–1^ than at 12 h^–1^, consistent with the longer residence time and the larger fraction of available O_3_ reacting under the lower-ACR conditions. However, this did not translate into a systematic dependence of molar product yields on ACR, as discussed below. In contrast, elevated RH had a clearer effect on product distribution: the formation of the volatile carbonyl products 4OPA and 6MHO increased at 70% RH compared with 20% RH. This is consistent with earlier studies showing that higher humidity enhances the formation or release of volatile carbonyl products from squalene ozonolysis.^3,7^ As shown in Table S5 (SI), the concentrations of 4OPA and 6MHO were approximately three times higher at the higher RH in the corresponding experiments, again in line with the previously reported humidity effects.^7^

For all tested scenarios, the highest proportion of available O_3_ reacted at the RH of 70% and the ACR of 6 h^–1^. To elaborate, since the concentration of O_3_ in the inlet flow was kept constant, the volume of air, and thus the moles of O_3_ passing through the chamber, was twice as large at the ACR of 12 h^–1^ as at 6 h^–1^. In relative terms, a smaller fraction of O_3_ reacts at 12 h^–1^ than at 6 h^–1^.

As shown in Figure 2, the skin oil constituents deposited on the plates for a week prior to the experiments were not being completely consumed during exposure to O_3_, with the concentration of their products in the chamber reaching a steady state after ∼40 min, except for the secondary product 4OPA, the concentration of which took ∼120 min to plateau. Ozone concentration reached steady-state in the first 15–20 min of the experiment. Conversely, in the case of the plates soiled through dermal contact (Figure 3), the ozone reactive skin oil constituents are almost completely consumed during the exposure interval for all combinations of RH and ACR, with outlet O_3_ slowly increasing during the time course of the experiment but without reaching an obvious steady-state concentration. The amount of O_3_ introduced in the 12 h^–1^ scenarios was double that introduced in the 6 h^–1^ scenarios. Consistent with that difference, at 12 h^–1^ the acetone concentrations peaked at 15 to 25 min from the beginning of the experiment, while at 6 h^–1^ this occurred at ∼40–45 min into the experiment. Acetone emerged as one of the dominant gas-phase products in our experiments, in line with chamber studies of skin-lipid ozonolysis.^5^ However, Morrison et al.^22^ noted that acetone’s contribution to volatile products of ozonolysis is difficult to quantify due to its high and variable indoor background levels. The peak concentrations of 6MHO and GA were larger at 70% RH than at 20% RH. The peak concentration of 4OPA, a terminal product produced exclusively by secondary and tertiary chemistry,^4,5,16^ appeared later, and at higher concentrations at elevated humidity. After 120 min at 6 h^–1^, the 4OPA mixing ratio was significantly larger than that of 6MHO (Figure 2a and 2c; Figure 3a and 3c). In contrast, at 12 h^–1^, the 4OPA mixing ratio was similar to that of 6MHO (Figure 2b and 2d; Figure 3b). This likely reflects the longer residence time available for gas-phase chemistry and 4OPA formation at 6 h^–1^ compared with 12 h^–1^. The mixing ratio profiles of the target volatile products from freshly soiled plates resemble more closely those observed for soiled t-shirts in our previous work^16^ than those for the plates soiled through dermal contact in this study.

Notably, the surfaces soiled by contact transfer of skin oil contained initially less squalene than freshly soiled surfaces, but they produced more than an order of magnitude higher volatile product mixing ratios. The concentrations of the gaseous and surface-bound squalene ozonolysis products (acetone, 4OPA, 6MHO and geranyl acetone) were normalized either to the amount of squalene consumed, Δsqualene (Table S7, SI), or to the initial amount of squalene on the surfaces (Figure S11, SI). The sum of the gaseous oxygenated VOC product amount ratios, related to the amount of squalene on the surface prior to oxidation, was 1.50 ± 0.28 in the “Dermal contact” scenario (excluding the anomalous experiment at 70% RH and ACR 12 h^–^1), and 0.16 ± 0.081 for the “Fresh” scenario. Further, the blank-corrected ΔO_3_/Δsqualene molar ratio was 2.27 ± 0.34 for the freshly soiled plates and 5.35 ± 0.61 for the dermally soiled plates, excluding the anomalous Dermal contact, 70% RH, 12 h^–1^ experiment. Since the symmetrical squalene molecule contains six C=C double bonds, in equivalent positions 1 and 1′, 2 and 2′, and 3 and 3′, up to six ozone molecules can react with one molecule of squalene. The lower squalene-normalized gas-phase product amount and lower blank-corrected ΔO_3_/Δsqualene molar ratio observed for the freshly soiled plates may indicate that squalene on surfaces soiled by direct dermal contact was more available to react with ozone.

Overall, based on the blank-corrected ozone loss used in the yield calculations, approximately 64–94 nmol of O_3_ reacted with freshly soiled plates and 140–183 nmol with dermal-contact plates, depending on RH and ACR. It should be noted that ozone loss reflects not only the six double bonds of squalene but also those of unsaturated fatty acids and triglycerides present in skin oil.^4,23^ The observed products, i.e., acetone, 6MHO, GA, and 4OPA, are consistent with previous laboratory studies^3–5,7,16^ and with observations of occupant-derived emissions.^22,24,25^ These results confirm that volatile ozonolysis products from skin oils can form rapidly indoors, with yields modulated by soiling mode, relative humidity, and air change rate.

### 3.3 Reaction Products Remaining on the Surface

The amounts of reaction products that remained on the glass plates are shown in Table S8 in the SI, expressed as total nanomoles per plate. On the vertically soiled surfaces, 6MHO and GA were below their respective LODs. Similarly, 6MHO was mostly below the LOD on the surfaces of dermal-contact plates, while GA was highest and most consistently quantifiable on freshly soiled plates.

On the freshly soiled plates, 38 and 40 nmol of squalene were consumed at 6 h^–1^ (65% and 69% of the initial amount), compared to 33 and 28 nmol at 12 h^–1^ (30% and 39%, respectively), although the amount of O_3_ introduced in the 12 h^–1^ scenarios was double that introduced in the 6 h^–1^ scenarios. For dermal-contact plates, ∼30 nmol of squalene (∼95% or more) were consumed across all RH and ACR combinations. These results suggest that nearly all squalene on the dermal-contact plates was depleted before the end of the exposure period (as can indeed be inferred from Figure 3), yielding a lower integrated ratio of moles of squalene consumed per mole of O_3_ consumed over ∼120 min. In addition, more O_3_ was consumed per mole of double bonds in skin oil at 70% RH than at 20% RH, indicating enhanced oxidative efficiency under humid conditions.

While squalene on the plates soiled through dermal contact was almost completely consumed during ozone exposure, only small amounts of the targeted reaction products were retained on the surface. Presumably, substantial amounts of nontargeted higher molecular weight primary products may have been retained on the surfaces; likely candidates include C_17_-trienal, C_22_-tetraenal, and C_27_-pentaenal.^5^ A skin wipe sample collected from Subject 2’s forehead was used to determine retention times and identities of a number of the nontargeted species. The C_17_, C_22_, and C_27_ unsaturated aldehydes were detected and confirmed against their reference mass spectra in the NIST library. They were subsequently quantified (Table S9, SI) in squalene equivalents (LOD = 0.08 nanomol/plate) and corresponded to only a few percent (2–3%) of squalene. The chromatograms from the analysis of the wipe samples from the “Fresh” and “Dermal contact” ozonation experiments were then searched using retention times and the “extract ion” procedure. Only C_22_-tetraenal could be detected and quantified in the samples from the freshly soiled plates. In the samples from the surfaces soiled by the dermal contact, C_22_-tetraenal was measured but could not be reliably quantified (below the LOD). C_17_-trienal was not detected, and C_27_-pentaenal likely coeluted with another compound and could not be distinguished from it. Given that these large unsaturated aldehydes represented a small fraction of the analyzed compounds in the actual skin sample, their nondetection in the samples wiped from the plates is not surprising.

### 3.4 Yields of the Gaseous and Surface-Bound Products

The molar yields of gas- and surface-phase products relative to ozone loss were calculated for each condition (Figures 4 and 5). The difference between the *noplate* and *plate* ozone concentrations (ΔO_3_,_reacted_) was corrected for blank chamber losses, including uptake to the desiccator walls and the glass armature, following eq 2:

2 $$Yield=\frac{C_{product}}{\Delta O_{3reacted}}$$

where C_product_ is the nanomoles of the compound formed and ΔO_3reacted._ is the blank-corrected nanomoles of ozone consumed.

**Figure 4:**
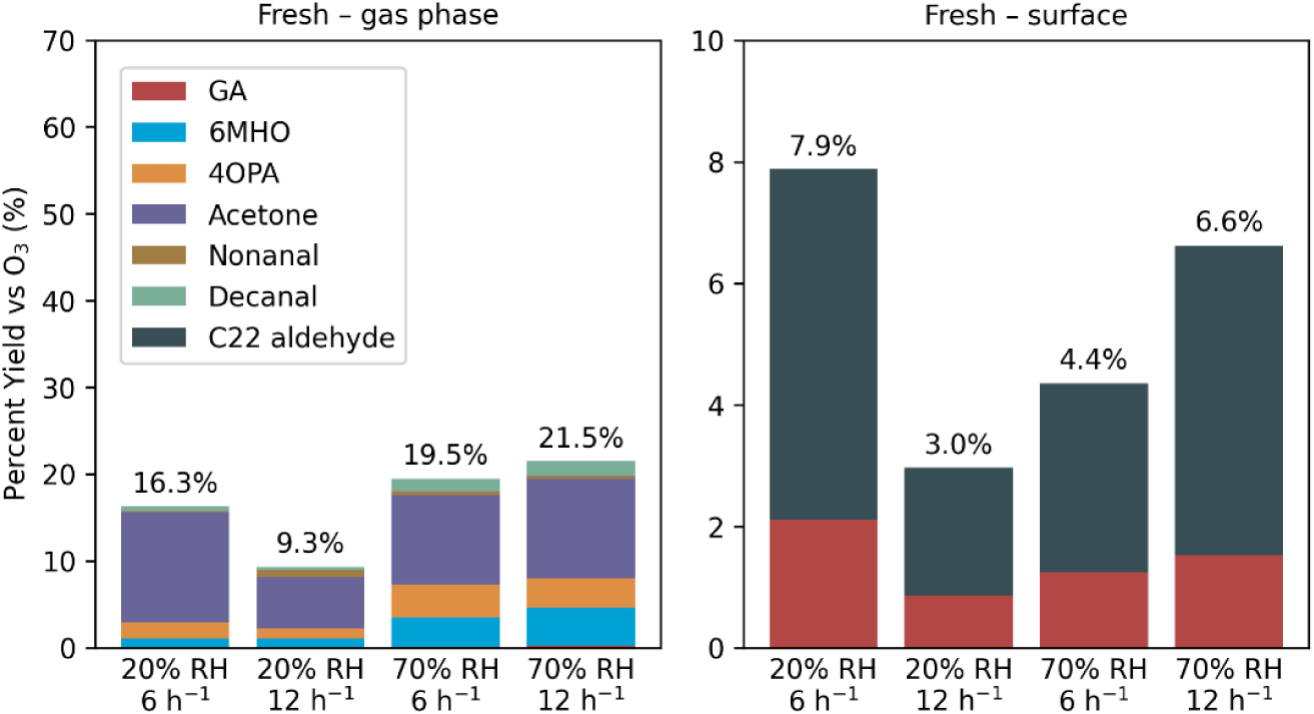
Yields of reaction products in the gas phase (left) and on surfaces (right) with respect to blank-corrected ΔO_3_ from freshly soiled plates, measured under different RH and ACR scenarios.

**Figure 5:**
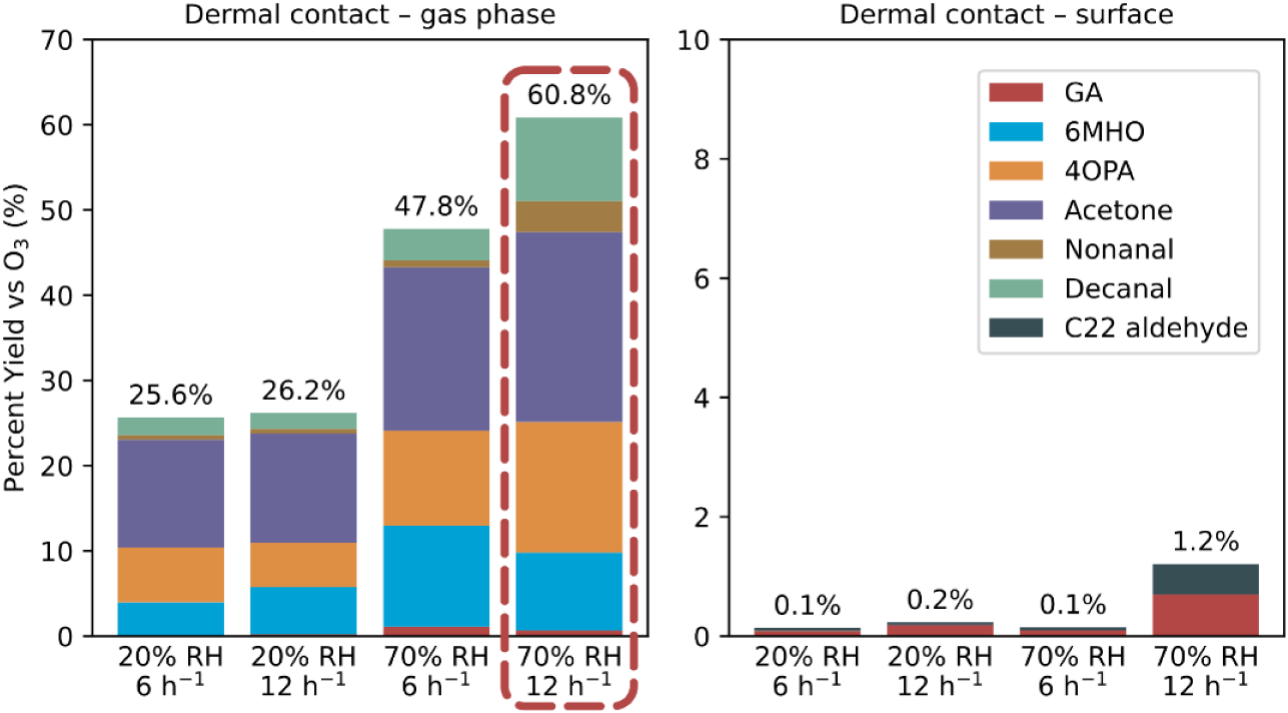
Yields of reaction products in the gas phase (left) and on surfaces (right) with respect to blank-corrected ΔO_3_ from plates soiled through dermal contact and measured under different RH and ACR scenarios. The *y*-axes are scaled to match Figure 4. Note that the yields shown for the gas-phase measurement at 70% RH, 12 h^–1^ are almost certainly the result of experimental error.

Among the quantified products, acetone dominated the gas-phase yields, with 6MHO and 4OPA being more dominant in the dermal contact scenario than in freshly soiled samples. The gaseous yields were higher for the dermally soiled surfaces compared to the freshly soiled surfaces, while the opposite was the case for the surface yields. Ventilation rate did not have a substantial effect on the gaseous product yields, as was also previously observed in ozone exposure experiments with human volunteers in a simulated aircraft cabin, at ventilation rates not so different from our conditions—ACR of 4.4 h^–1^ and 8.8 h^–1^, respectively.^26^ The total yield for the freshly soiled surface at 20% RH and ACR 6 h^–1^ (16.3%) is somewhat higher than anticipated due to the higher yield of acetone. We cannot provide a straightforward explanation and therefore retained the measured values. Based on the duplicate measurements, the sampling process was a major contributor to the estimated uncertainty budget. Dermal-contact plates exhibited higher absolute gas-phase product concentrations (Figures 2 and 3), consistent with their greater total ozone uptake assessed by the molar ozone/squalene ratio of ∼5 vs the corresponding value of ∼2.5 for the freshly soiled plates. Notably, almost all squalene was consumed on the dermally soiled surfaces, whereas approximately half of the initially available squalene remained unreacted on the freshly soiled surfaces (Table 3). The higher molar ratio of ozone/squalene may have also contributed to an increase of secondary products (e.g., 4OPA) relative to primary products (6MHO, GA).

The surface yields do not show any systematic effect of either RH or ACR. Although we provide the amounts of nanomol/plate of 6MHO analyzed, we do not anticipate substantial amounts of 6MHO to remain on the surface as a product from the reaction in the chamber. The measured 6MHO was likely present in the bulk of the squames and released during extraction. For this reason, it is not included in Figures 4 and 5. The magnitude of the surface product yields was mainly affected by the mode of soiling, with substantially larger yields from the freshly soiled surfaces. However, we cannot rule out that some amounts of geranyl acetone and C_22_-aldehyde were already present in the squames on the freshly soiled plates even prior to the ozone reaction in the chamber.

The analyzed surface concentrations of nonanal and decanal from the freshly soiled plates were surprisingly high, particularly at 70% RH and 6 h^–1^ (Table S8, SI). Given the volatility of these compounds, we found it unrealistic that they have been formed in the ozonolysis of skin oils in the experiments. A possible explanation for the aldehydes being detected in such large amounts on the plates may be the soiling process itself, as the aldehydes may have been present in the depositing organic material containing skin flakes, or from other indoor sources in the office and home environment, or the slept-in t-shirts. Freshly deposited squames may also already contain products of earlier ozonolysis reactions.^27^ For this reason, we have chosen not to include nonanal and decanal in the surface yield considerations (Figures 4 and 5).

It should be noted that one result for the dermal contact scenario, the yields shown for the gas phase measurement at 70% RH, 12 h^–1^, seemingly fit the trend of higher yields at higher RH, but we still consider this experiment as faulty. The Δsqualene for that sample is consistent with other measurements in that series (Table 3) as are the relative yields vs ozone. However, both Δozone and the absolute VOCs mixing ratios are very low compared to the other samples (Figure 3). This would be consistent with a leak somewhere in the chamber. It seems like the reaction of ozone with the skin oil-soiled plates proceeded similarly to the other samples (ozone consumed), but the gas-phase measurements were affected by unknown factors.

Figure 6a shows a direct comparison between our gas-phase yields resulting from reaction with O_3_ and a recent study,^28^ which also examined ozone reaction with controlled indoor-relevant surfaces, including glass surfaces contaminated with aged indoor films, pure squalene, and skin oil. Following the terminology used by Downey and Abbatt,^28^ these are referred to as differential yields, distinguishing them from the total yields compiled in Figure 6b. The squalene and skin-oil experiments reported by Downey and Abbatt,^28^ i.e., glass surfaces freshly coated with squalene or contacted with a volunteer’s hands, are most directly comparable to the “dermal-contact” scenario in this study. In this context, it is notable that the skin-oil yield measured at 45% RH falls between our dermal-contact yields at 20% and 70% RH, consistent with the positive RH dependence observed in our experiments. The referenced “Bedroom” experiment is conceptually closer to the “Fresh” scenario in this study, as both represent occupant-influenced indoor surface films rather than freshly applied model compounds. However, the “Bedroom” yield in Downey and Abbatt was dominated by nonanal, which made only a minor contribution in our “Fresh” samples. This likely reflects differences in the composition and history of the deposited films, as the authors attributed high nonanal from apartment-aged surfaces largely to oleic acid/triolein-type precursors associated with cooking oils or related indoor SVOCs, while our “Fresh” plates were primarily home- and office-soiled surfaces enriched with squames from slept-in textiles, and therefore appear more strongly influenced by skin-lipid/squalene chemistry than by cooking-oil-derived unsaturated lipids.^22,28^

**Figure 6:**
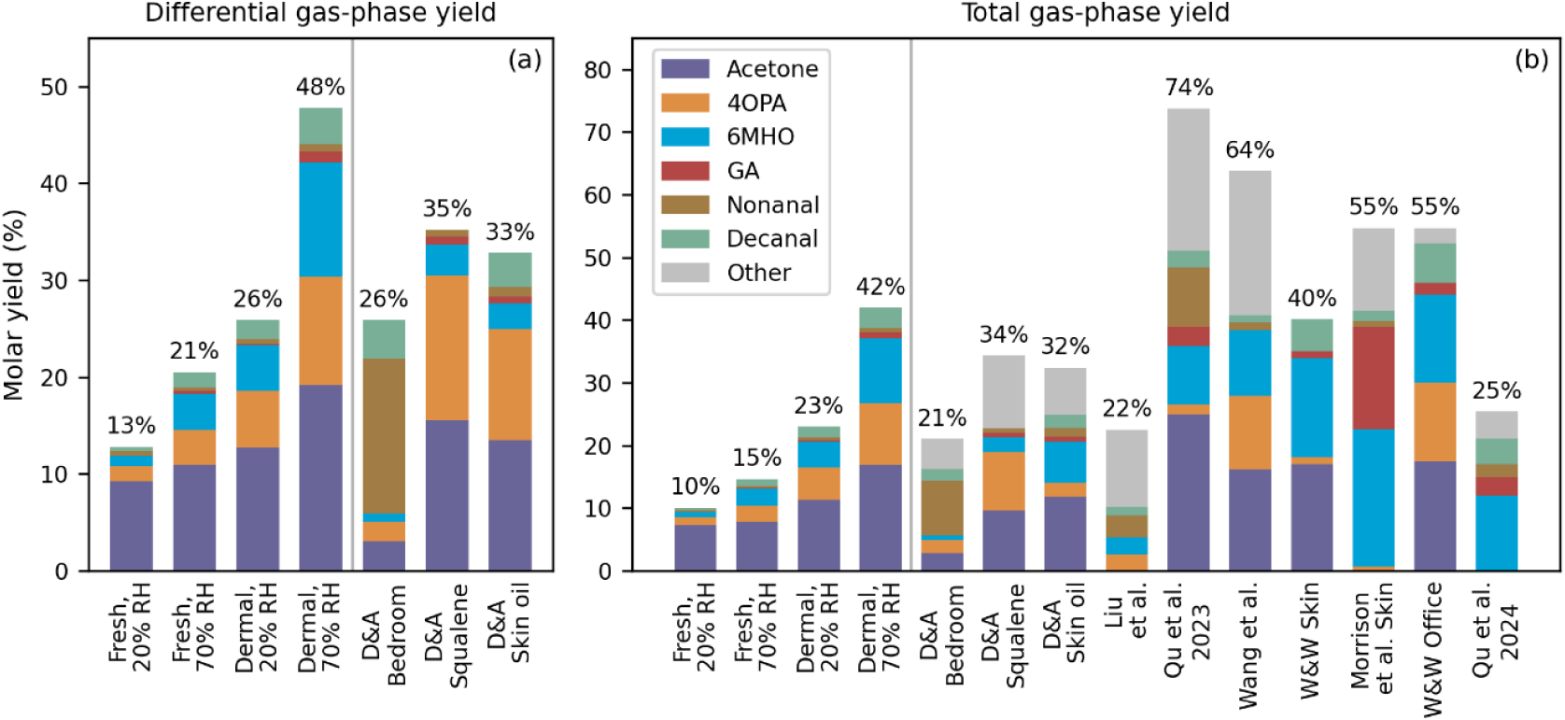
(a) Differential yields for the present study and selected surface experiments, following the terminology used by Downey and Abbatt (D&A).^28^ Only compounds quantified in the present study are shown. The “Fresh” and “Dermal” (Dermal contact) values are RH-specific means, with the faulty dermal-contact 70% RH, 12 h^–1^ experiment excluded. “D&A Bedroom”, “D&A Squalene”, and “D&A Skin oil” refer to aged-bedroom-surface, squalene-coated glass, and skin-oil-contaminated glass experiments, respectively, as reported in D&A.^28^ (b) Total yields (OVOC formation divided by total O_3_ loss) from the present study and selected literature studies: “Liu et al.”—an occupied-residence study;^29^ “Qu et al. 2023”, “Wang et al.”—human chamber emission experiments;^30,31^ “W&W (Wisthaler & Weschler) Skin” and “W&W Office”—human skin/office experiments;^5^ “Morrison et al. Skin”—direct bare-skin flux-cell measurements;^22^ “Qu et al. 2024”—office study.^32^ In subplot (b), quantified products not measured in the present work are grouped as “Other” where applicable. Some underlying values in subplot (b) were very kindly provided by Professor Jonathan Abbatt and Ms. Jillian P. Downey, the authors of D&A.^28^

Figure 6b places the present results in a broader literature context using total gas-phase yields. The compiled studies include controlled surface experiments, direct skin measurements, human chamber experiments, and occupied indoor environments.^5,22,29–32^ The “Fresh” scenario lies near the lower end of the reported range, although the comparison is conservative because the present work targeted fewer OVOCs than some of the literature studies. The dermal-contact scenario is closer to skin-oil and human-occupant studies, especially when considering the shared squalene ozonolysis products. The high 6MHO and GA but comparatively low 4OPA contribution reported by Morrison et al.^22^ is consistent with their direct bare-skin, short-residence-time flux-cell design, which favors primary ozonolysis products and limits secondary oxidation before measurement. In contrast, whole-body and room-scale studies can include longer residence times, clothing effects, transferred skin oils, and gas-phase processing, which may enhance secondary products such as 4OPA.^30–32^

### 3.5 Main Takeaways

The mode of soiling was the dominant factor impacting ozone uptake and product formation. Plates soiled through dermal contact consistently produced the highest gas-phase product concentrations (under comparable O_3_ exposure conditions), followed by freshly soiled plates, horizontally soiled (for months) plates, and vertically soiled (for months) plates. Despite initially containing less squalene than the freshly soiled plates, dermal-contact plates yielded more than an order of magnitude higher concentrations of volatile products. This could be due to the more homogeneous, continuous films produced by direct transfer in contrast to the more heterogeneous deposition associated with freshly deposited squames, or to a difference in the chemical composition of the samples produced using different soiling modes, including nontarget compounds. The films accumulated over months on the long-term soiled plates were less reactive, and also are anticipated to have reacted to some extent with room ozone and hydroxyl radicals during the months that they were exposed to room air.

Within each soiling type, environmental parameters further affected the chemistry. Elevated RH promoted the formation of volatile carbonyl products, with higher molar yields of 6MHO and 4OPA observed at 70% RH than at 20% RH. In contrast, ACR did not produce a systematic change in the molar yields of the gaseous ozone-reaction products, although lower ACR increased residence time and, in some cases, the integrated gas-phase product amounts and fractional ozone uptake. The yields of the surface-bound products did not systematically vary with either RH or ACR.

The results of this study demonstrate that ozone removal and product formation are governed not simply by the total surface loading of skin oil, but by its deposition mode, the accessibility of reactive constituents within the film, and the surrounding environmental conditions (i.e., RH and ventilation rate). In real-life environments, contact transfer from skin or clothing is likely to be a more consequential source of reactive surface films than passive accumulation of squames or aged films, with implications for ozone loss and product emissions near high-contact surfaces such as desktops, chairs, keyboards, and phones. This highlights the need to account for both film morphology and environmental drivers when assessing occupant-derived contributions to indoor ozone chemistry.

## Supplementary Material



## Notes

Additional experimental details; methods; plots; photographs of the experimental setup and sampling (PDF)
