# Supplementary material for: Ozone Reactions with Indoor Surfaces Soiled by Human Skin Oil are Impacted by Manner of Soiling, Relative Humidity, and Air Change Rate
Source: Environ Sci Technol. 2026 Jul 30;60(31):21837–48. doi: 10.1021/acs.est.6c09797 (PMC13472148; doi:10.1021/acs.est.6c09797)
Supplement: Supplementary file 1 [file es6c09797_si_001.pdf]

Supporting Information for: Ozone reactions with indoor surfaces soiled by human skin oil are impacted by manner of soiling, relative humidity, and air change rate

Authors: Wojciech Wojnowski[1,2*], Armin Wisthaler[2], Tomas Mikoviny[2], Gabriel Bekö[3], Charles J. Weschler[3,4], Ann Sjöblom[5], and Sarka Langer[5,6]

[1]Gdańsk University of Technology, Faculty of Chemistry, Department of Analytical Chemistry, 80-233 Gdańsk, Poland
[2]University of Oslo, Department of Chemistry, N-0315 Oslo, Norway
[3]International Centre for Indoor Environment and Energy, Department of Environmental and Resource Engineering, Technical University of Denmark, DK-2800 Lyngby, Denmark
[4]Environmental and Occupational Health Sciences Institute, Rutgers University, Piscataway, New Jersey 08854, United States
[5]IVL Swedish Environmental Research Institute, Environmental Chemistry, SE-400 14 Göteborg, Sweden
[6]Chalmers University of Technology, Department of Architecture and Civil Engineering, Division Building Services Engineering, 412 96 Göteborg, Sweden
wojciech.wojnowski@pg.edu.pl

- # of figures: 12
- # of tables: 9
- # of supplementary texts: 3

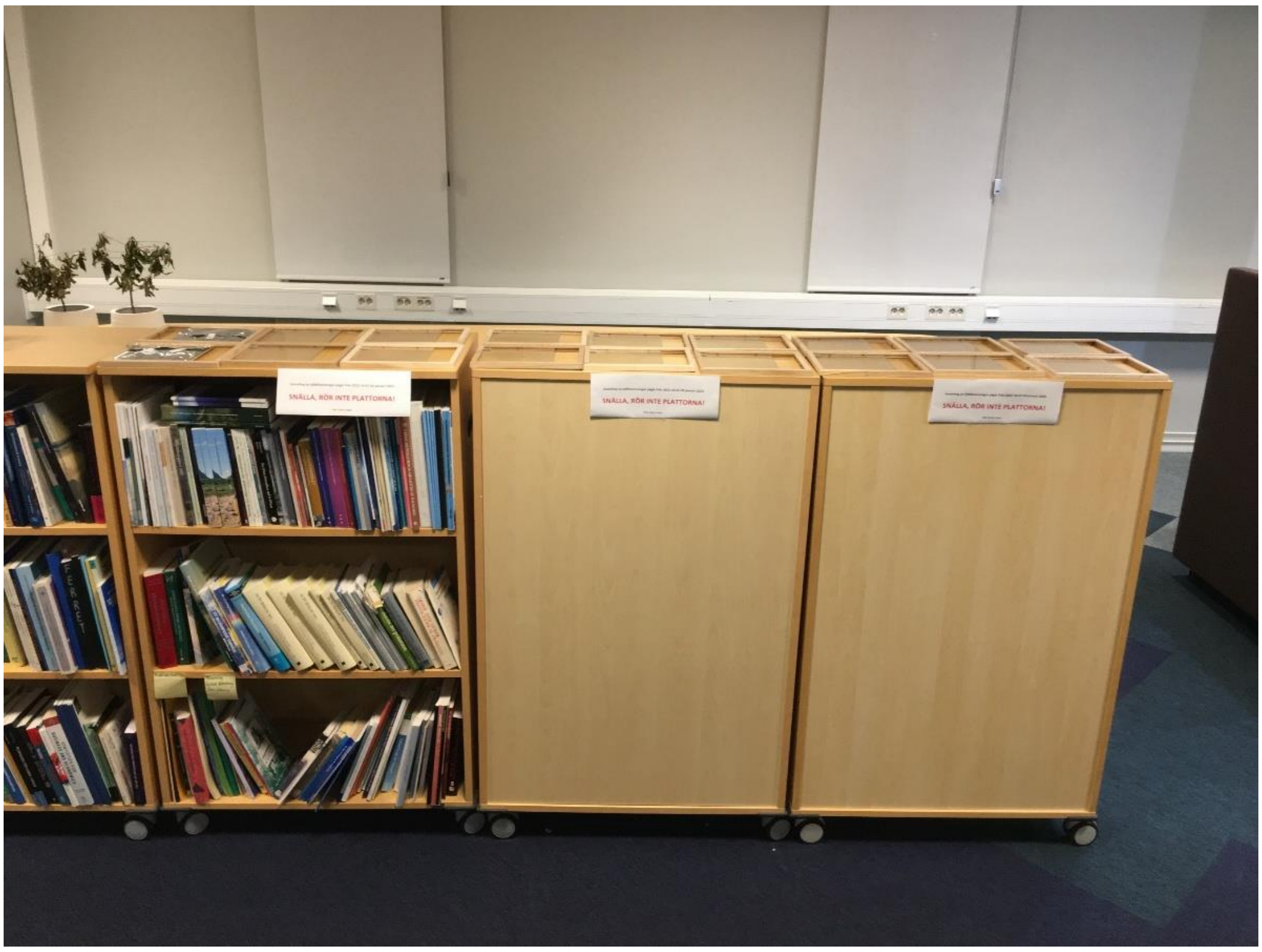

Figure S1. Plates soiled in the horizontal position. The sign says: “Do not touch the plates, please”.

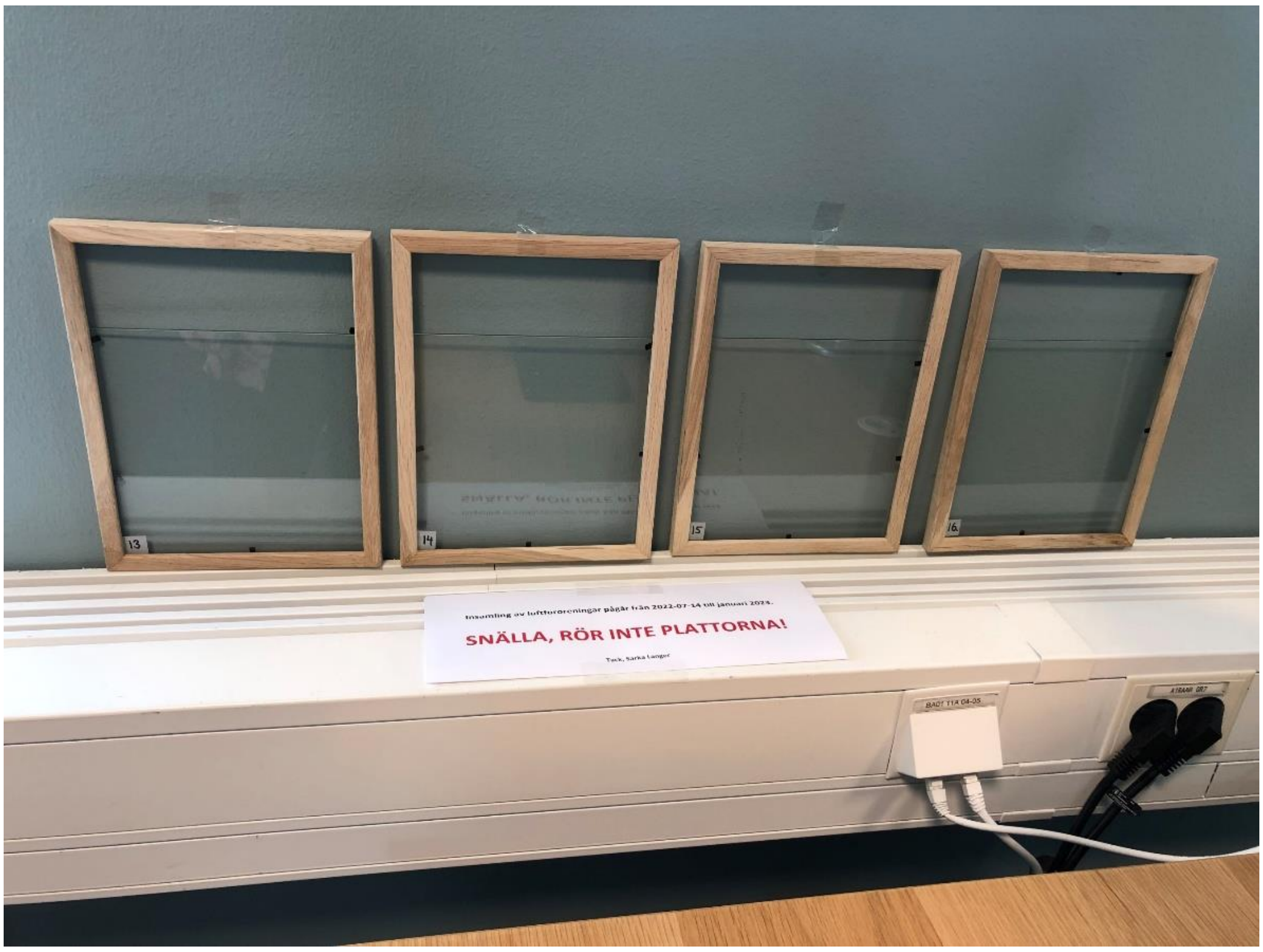

Figure S2. Plates soiled in the vertical position. The sign says: “Do not touch the plates, please”.

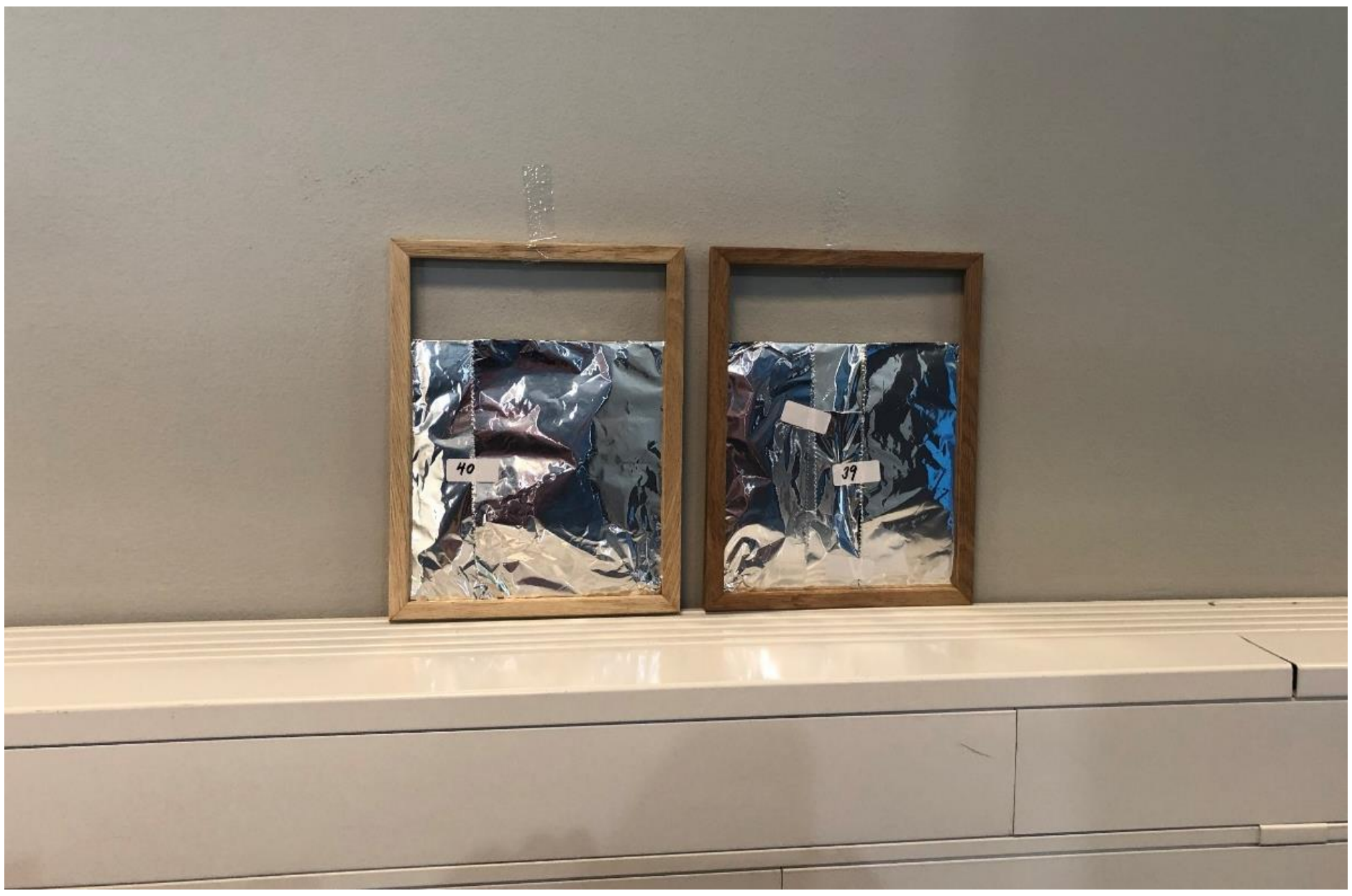

Figure S3. Plates intended as blank samples, covered with aluminium foil.

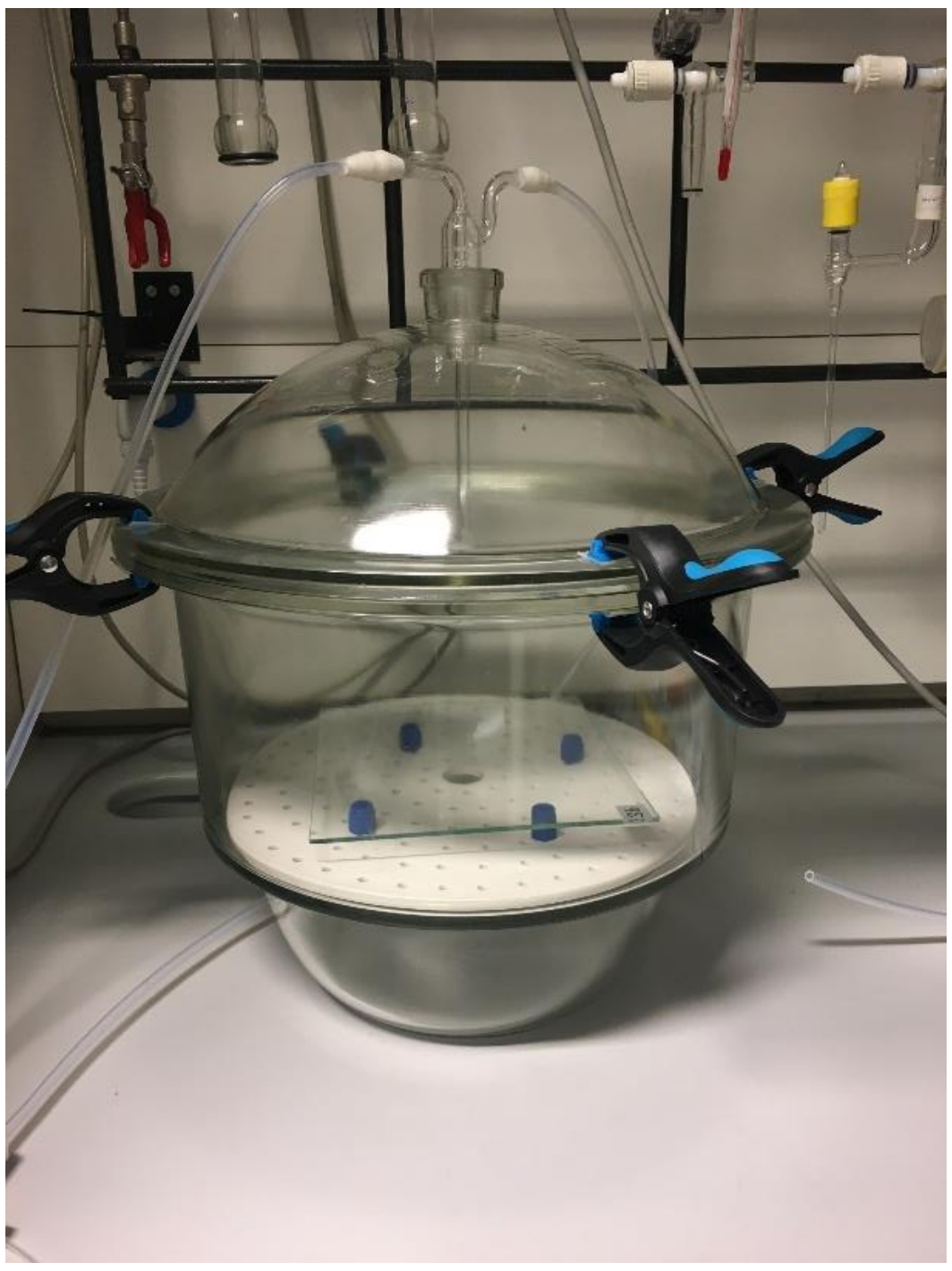

Figure S4. The desiccator with inlet and outlet gas lines and the glass plates in the experimental setup.

Table S1. Temp and RH during the soiling periods.

Soiling position	T (°C)	RH (%)	Soiling start	Soiling end
Horizontal	21.8 ± 0.45	32 ±11	2022-10-06	2023-01-25
Vertical	21.2 ± 0.59	45 ±13	2022-07-14	2023-01-25

Supporting Text S1. Chemical analysis of the surface wipes

The samples were spiked prior to extraction with isotopically labelled internal standard diethyl hexyl phthalate-d4 (50 µg per sample). The spiked samples were equilibrated for 30 min at room temperature and then extracted in an ultrasonic bath for 2 x 30 minutes using 2 × 15 mL of extraction solvent, consisting of a mix of pentane:hexane 2:1 by volume with 6 % diethyl ether according to the procedure originally described elsewhere.[1]

The extracts from the subsequent extractions were pooled and dried under a gentle stream of nitrogen at room temperature to approximately 1 mL. The exact volume of the extracts was determined by weighing, using the density of hexane (0.655 g cm^{-3}) assuming that pentane and diethyl ether evaporated during the preconcentration step. The extracts were then transferred to vials and 10 µg of the injection standard squalane was added prior to the instrumental analysis.

The analyses were carried out using gas chromatography with mass spectrometry (GC/MS) as the detection technique on a TRACE 1310 GC System coupled to a MS – TSQ 8000 evo (Thermo Fisher Scientific) in a Selected Ion Monitoring (SIM) mode with electron impact ionization source (EI). The GC was operated in Splitless with Surge mode with an inlet temperature of 275 °C. Two microliters were injected using a Thermo Triplus 100LS auto injector onto a TG-5SILMS column (length 30 m x diameter 0.25 mm x film thickness 0.25 µm). The initial oven temperature of 50 °C was held for 2 min, raised to 320 °C at 6 °C min^{-1} and then held for 13 min (62 min in total). Chromeleon software was used for instrument control, data acquisition and evaluation.

Quality assurance and quality control. The calibration curves for all (but one) compounds in Table S2 were linear in their ranges of 1 - 40 ng μL^{-1}, with the linearity coefficients $R^2 \geq 0.99$. The extraction efficiencies were evaluated from the amounts of the spiked recovery standard diethyl hexyl phthalate-d_4 (0.97 ± 0.06). The extraction efficiency of squalene of 1.04 ± 0.06 for the analytical procedure was determined in our previous work.[2] Concentrations in the clean gauze pads used as procedural blanks, detection limits, and reproducibility of replicate injections of selected compounds are presented in Table S2. The detection limits were calculated as 3 × standard deviation in the procedural blanks. A quadratic calibration curve[3] was generated for pentadecanoic acid in the range of 0 – 23 ng μL^{-1} ($R^2 = 0.99$). The detection limit of the pentadecanoic acid of 1.5 µg $sample^{-1}$ (6.2 nanomole/sample) was determined from the intercept of the calibration curve.

---

[1] Rudel, R.A.; Camann, D.E.; Spengler, J.D.; Korn, L.R.; Brody, J.G. Phthalates, alkylphenols, pesticides, polybrominated diphenyl ethers, and other endocrine disrupting compounds in indoor air and dust. *Environ. Sci. Technol*. **2003**, 37*,* 4543-455.

[2] Langer, S., Weschler, C.J., Bekö, G., Morrison, G., Giovaloulis, G., Wargocki, P., Wang, N., Zannoni, N., Yang, S., Williams, J. Squalene depletion in skin following ozone exposure. *Environ. Sci. Technol*. **2024**, 58*,* 6693-6703.

[3] Schwink, S.K.; Schmid, M.J.; Silberstein, J.M.; Vance, M.E. Significant Variability in Terpene Content and Secondary Organic Aerosol Formation Potential from Ozonolysis of Consumer Product Categories Used Indoors. *ACS EST Air* **2026**, 3, 981-989.

Table S2. Average and standard deviation of procedural blanks (n = 6) and detection limits (three times the standard deviation of the blanks) are in units of µg/sample (wipe), and LODs are also in nmole/sample. Analytical reproducibility and precision of replicates are in % of Relative Standard Deviation.

Compound	Average	STD	LOD µg	LOD nmole	RSD (%)
Squalene	0.031	0.011	0.03	0.08	4.4
6MHO	0.046	0.026	0.08	0.6	8.8
GA	0.015	0.011	0.03	0.2	9.3
Nonanal	0.037	0.005	0.02	0.1	6.1
Decanal	0.048	0.032	0.1	0.6	6.1

Table S3. Repeatability summary using duplicate measurements at 20% RH, 12 h^{-1} ACR. Replicate values for gas and surface target analytes given in nmole/sample. SD and RSD are shown as descriptive indicators only, because each summary is based on n=2 measurements. The last two columns contain the estimated relative uncertainties based on the main instrumental and analytical contributions relevant to each phase.

Phase/var	Soiling cond.	Target	Rep. 1	Rep. 2	Mean	SD	RSD (%)	U_{rel} vs O_3 (%)	U_{rel} vs sq. (%)
Reaction extent (%)	Fresh	Squalene reacted	30.13	29.4	29.76	0.36	1.21	-	-
		O_3 reacted	12.84	15.49	14.16	1.32	9.33	-	-
	Dermal contact	Squalene reacted	97.46	96.8	97.13	0.33	0.34	-	-
		O_3 reacted	23.91	29.69	26.8	2.89	10.8	-	-
Gas	Fresh	Acetone	4.56	5.48	5.02	0.46	9.2	6	16
		4OPA	0.84	1.14	0.99	0.15	15	13	25
		6MHO	0.73	1.00	0.87	0.13	15.4	14	26
		GA	0.04	0.04	0.04	<0.01	4.9	24	20
		Nonanal	0.69	0.67	0.68	0.01	1.8	18	10
		Decanal	0.28	0.37	0.33	0.05	14.1	12	24
	Dermal contact	Acetone	22.3	24.7	23.5	1.2	5.1	8	11
		4OPA	9.03	10.0	9.51	0.49	5.1	12	14
		6MHO	10.32	10.03	10.17	0.15	1.5	18	10
		GA	0.40	0.36	0.38	0.02	5.2	29	21
		Nonanal	0.92	1.16	1.04	0.12	11.5	11	21
		Decanal	2.89	3.82	3.36	0.47	13.9	12	24
Surface	Fresh	6MHO	1.72	1.78	1.75	0.03	1.6	15	11
		GA	0.7	0.76	0.73	0.03	4.3	12	11
		C22 aldehyde	1.96	1.57	1.76	0.19	10.9	30	17
		Nonanal	12.99	11.82	12.41	0.59	4.7	22	11
		Decanal	9.98	12.22	11.1	1.12	10.1	10	19
		C15 acid	88	119	103.6	15.3	14.8	13	25
	Dermal contact	6MHO	1.05	n.d.	1.05	n.a.	n.a.	10	10
		GA	0.37	0.32	0.34	0.03	7.3	25	13
		C22 aldehyde	<0.08	<0.08	<0.08	n.a.	n.a.	-	-
		Nonanal	2.42	3.44	2.93	0.51	17.3	15	28
		Decanal	8.65	11.66	10.15	1.5	14.8	13	25
		C15 acid	15	13	14	0.95	6.8	25	13

n.d. = not detected; n.a. = not applicable; <0.08 values were below the reporting threshold.

Supporting Text S2. Calculation of ozone deposition velocity and removal rate coefficients

The O_3 deposition velocity was calculated by slightly modifying the approach used by Yang *et al.* [4] (1):

$$v_d = \frac{V}{A}\lambda\frac{O_{3\,noplate} - O_{3\,plate}}{O_{3\,plate}} \tag{1}$$

where:

V – chamber volume (0.0195 m^3);
A – surface area of the plate (0.0324 m^2);
λ – air change rate (either 6 h^{-1} or 12 h^{-1});
$O_{3\ noplate}$, $O_{3\ plate}$ – ozone measured at the outlet with an empty chamber and when exposing the soiled plates, respectively.

Equation (1) was modified to correct for the O_3 loss in the empty chamber by calculating the background first-order rate constants (k_{bg}) for the blank measurements under matching experimental conditions (2,3):

$$k_{bg} = \lambda\frac{O_{3\,noplate,BLANK} - O_{3\,plate,BLANK}}{O_{3\,plate,BLANK}} \tag{2}$$

$$v_d = \frac{V}{A}\frac{\lambda\left(O_{3\,noplate,SOILED} - O_{3\,plate,SOILED}\right) - k_{bg}O_{3\,plate,SOILED}}{O_{3\,plate,SOILED}} \tag{3}$$

In the case of the freshly soiled samples, the steady-state deposition velocity was derived from the $v_d(t)$ of the last 30 min of the respective experiments, with the absolute value of $O_{3,\,out} \leq 0.02$ ppb min^{-1}.

The O_3 removal rate coefficients from the blank and soiled glass plates were calculated as follows (4,5):

$$k_{p_{BLANK}} = ACR\left(\frac{O_{3\,noplate_{BLANK}}}{O_{3\,plate_{BLANK}}} - 1\right) \tag{4}$$

$$k_{p_{SOILED}} = ACR\left(\frac{O_{3\,noplate_{SOILED}}}{O_{3\,plate_{SOILED}}} - \frac{O_{3\,noplate_{BLANK}}}{O_{3\,plate_{BLANK}}}\right) \tag{5}$$

where:

$k_{p\ BLANK}$ – ozone removal coefficient with the blank sample (clean plate) in the chamber
$k_{p\ SOILED}$ – net ozone removal coefficient with the sample (soiled plate) in the chamber
$O_{3\ noplate}$, $O_{3\ plate}$ – ozone measured at the outlet with an empty chamber and when exposing the soiled plates, respectively

[4] Yang, S.; Gao, K.; Yang, X. Volatile organic compounds (VOCs) formation due to interactions between ozone and skin-oiled clothing: Measurements by extraction-analysis-reaction method, *Build. Environ.* **2016**, 103, 146-154.

Table S4. Mixing ratios of ozone with an empty chamber and when exposing the soiled plates as measured by the downstream monitor.

Soiling condition	Scenario	$O_{3\ noplate}$ ppb	$O_{3\ plate}$ ppb	ΔO_3 ppb	$\Delta O_{3\ corr.}$ (ppb)	Average per soiling mode (ppb)
Blank	20% 6 h^{-1}	47.0	45.5	1.6		
Blank	20% 12h^{-1}	39.3	37.9	1.3		
Blank	70% 6 h^{-1}	33.0	31.3	1.7		
Blank	70% 12h^{-1}	31.0	29.3	1.7		
Vertical	20% 6 h^{-1}	48.6	47.0	1.7	0.1	
Vertical	20% 12h^{-1}	39.0	37.8	1.2	n.a.	
Vertical	70% 6 h^{-1}	38.8	35.7	3.1	1.4	Vertical
Vertical	70% 12h^{-1}	31.2	30.6	0.6	n.a.	0.75
Horizontal	20% 6 h^{-1}	48.2	44.5	3.7	2.1	
Horizontal	20% 12h^{-1}	38.3	35.1	3.2	1.9	
Horizontal	70% 6 h^{-1}	35.0	31.3	3.7	2	Horizontal
Horizontal	70% 12h^{-1}	31.7	30.4	1.2	-0.5	1.4
Fresh	20% 6 h^{-1}	42.7	34.6	8.0	6.4	
Fresh	20% 12h^{-1}	38.9	34.0	5.0	3.7	
Fresh duplicate	20% 12h^{-1}	38.8	32.8	6.0	4.7	
Fresh	70% 6 h^{-1}	37.6	30.1	7.4	5.7	Freshly soiled
Fresh	70% 12h^{-1}	33.2	28.3	4.9	3.2	4.3
Dermal contact	20% 6 h^{-1}	46.0	30.6	15.5	13.9	
Dermal contact	20% 12h^{-1}	37.1	28.3	8.9	7.6	
Dermal contact duplicate	20% 12h^{-1}	37.7	26.5	11.2	9.9	
Dermal contact	70% 6 h^{-1}	33.3	19.3	14.0	12.3	Dermal contact
Dermal contact	70% 12h^{-1}	27.9	25.5	2.5	0.8	7.7

n.a. = too low to measure reliably.

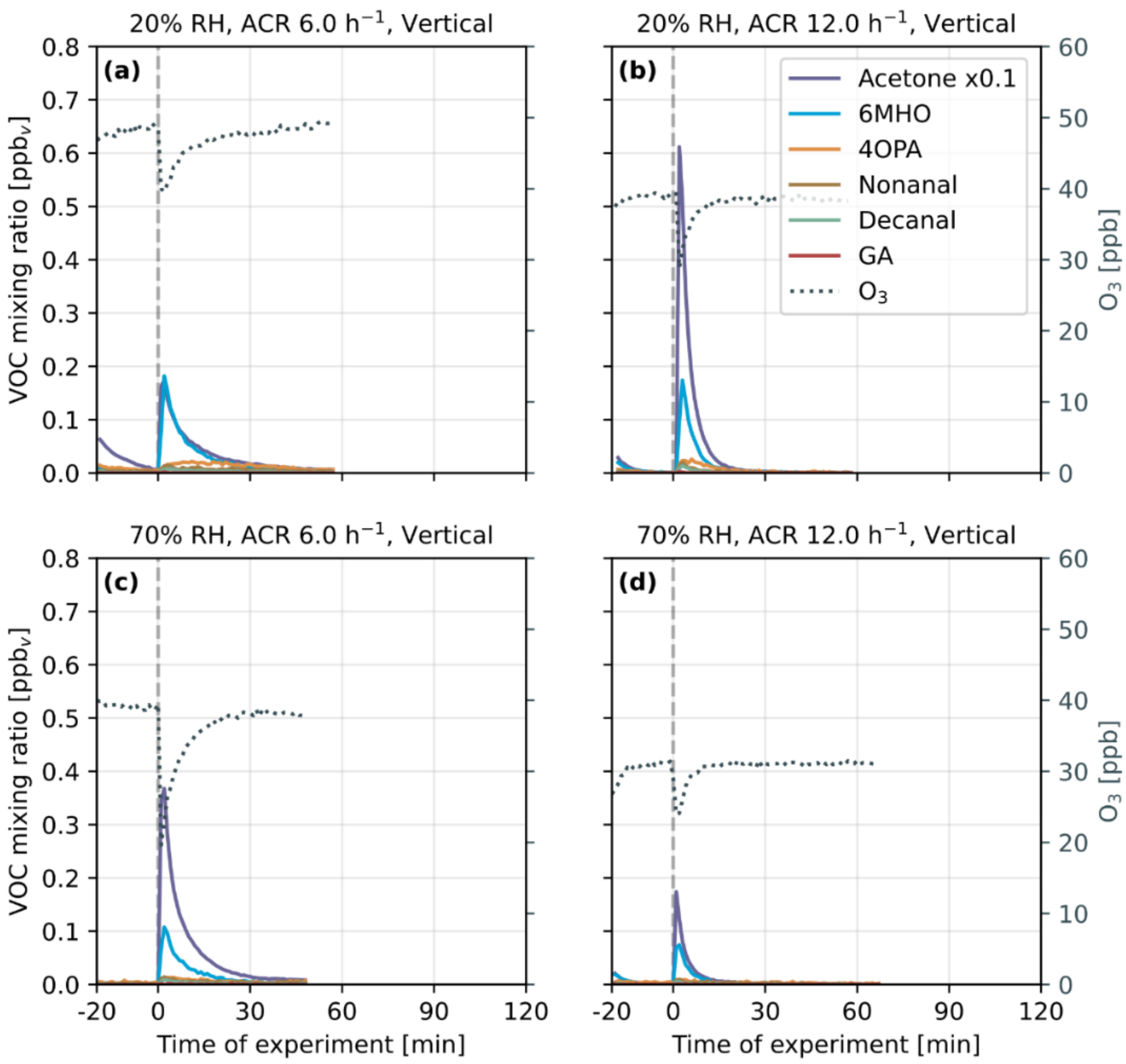

Figure S5. Mixing ratios of the monitored compounds for the duration of the experiments under different measurement conditions in the '**Vertical**' scenario and the corresponding O_3 mixing ratios. The signals include the 6MHO and acetone interference. Note the scaling of the acetone mixing ratios for clarity.

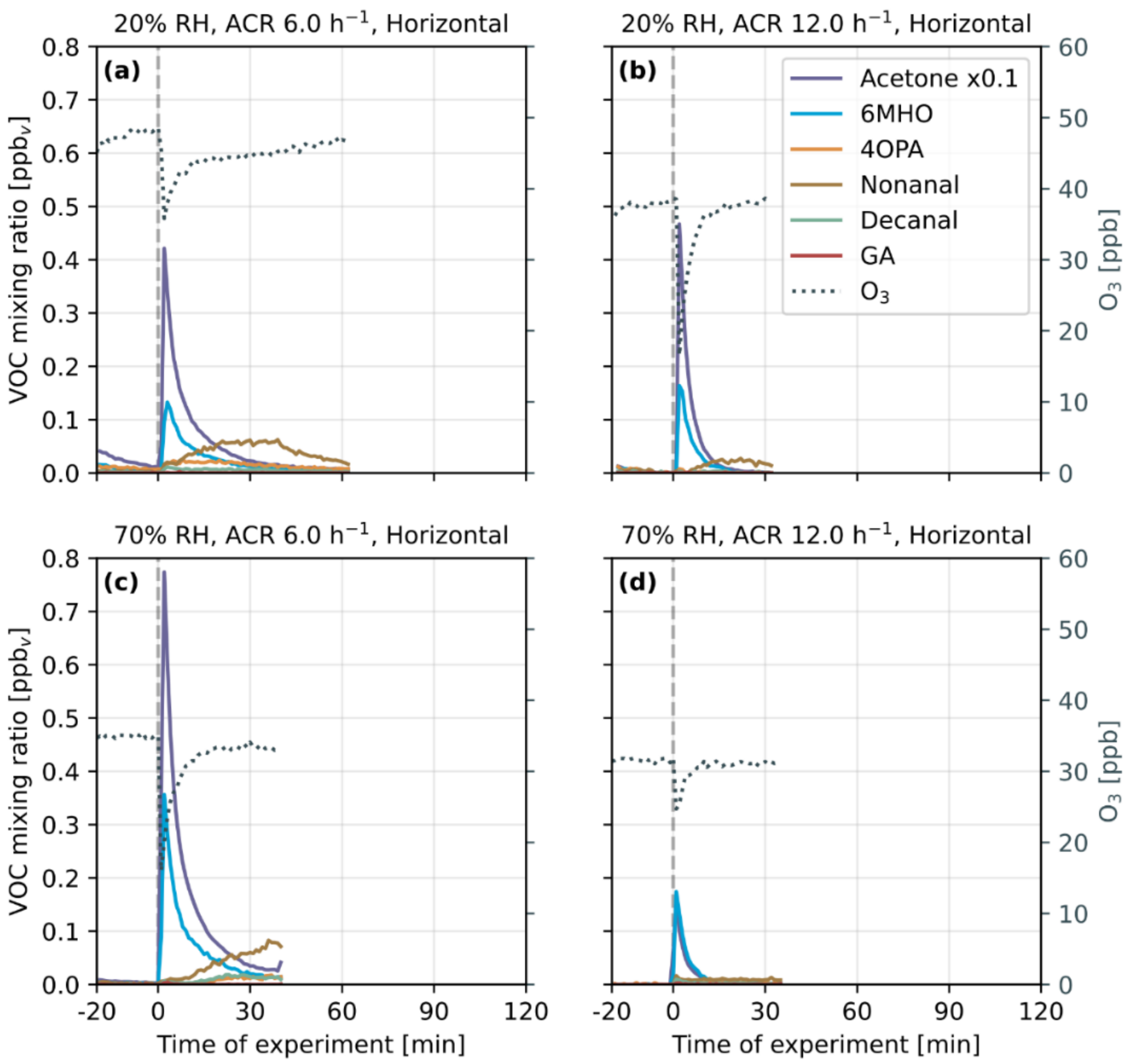

Figure S6. Mixing ratios of the monitored compounds for the duration of the experiments under different measurement conditions in the '**Horizontal**' scenario and the corresponding O_3 mixing ratios. The signals include the 6MHO and acetone interference. Note the scaling of the acetone mixing ratios for clarity.

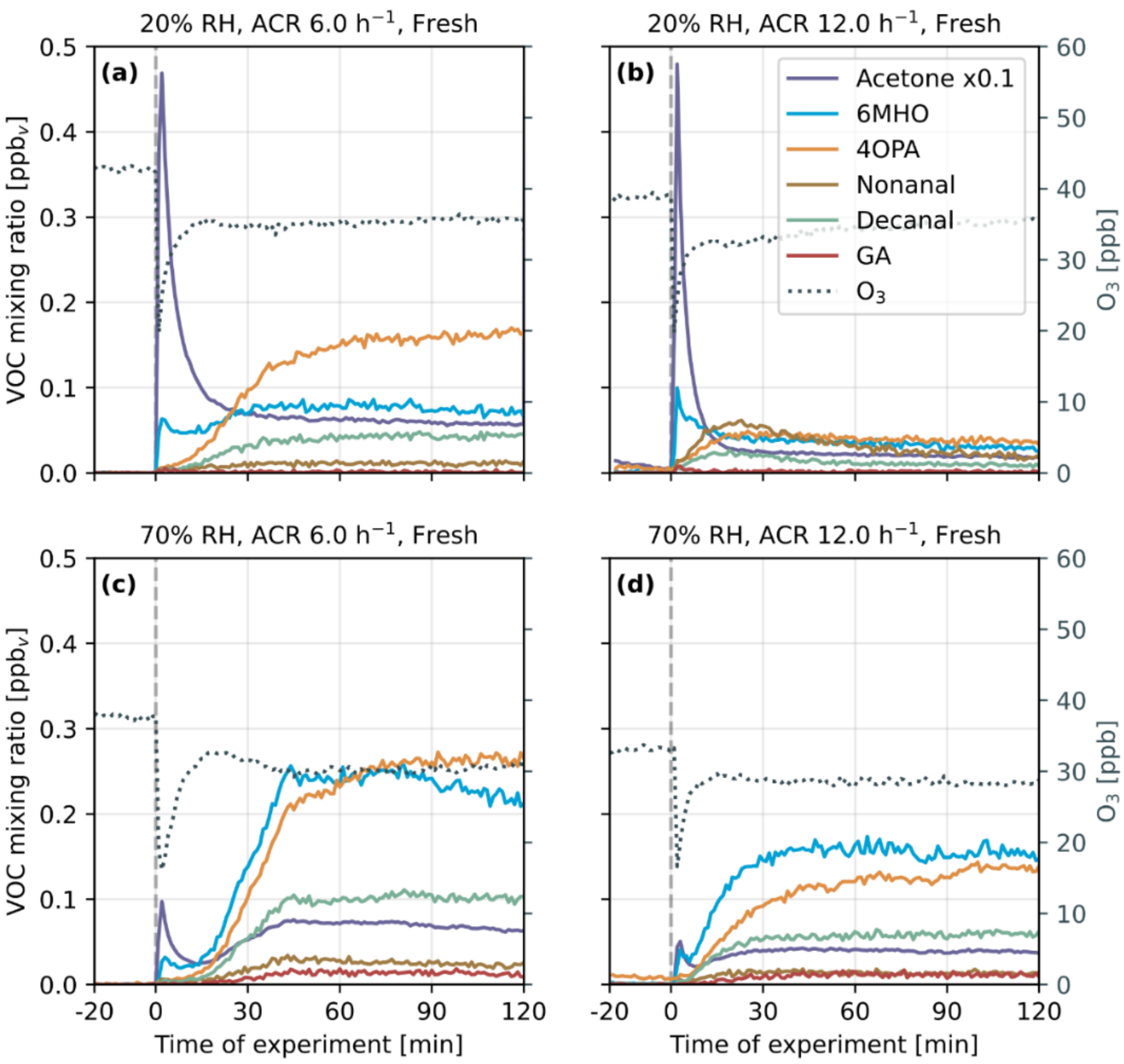

Figure S7. Mixing ratios of the monitored compounds for the duration of the experiments under different measurement conditions in the '**Fresh**' scenario and the corresponding O_3 mixing ratios. The signals include the 6MHO and acetone interference. Note the scaling of the acetone mixing ratios for clarity.

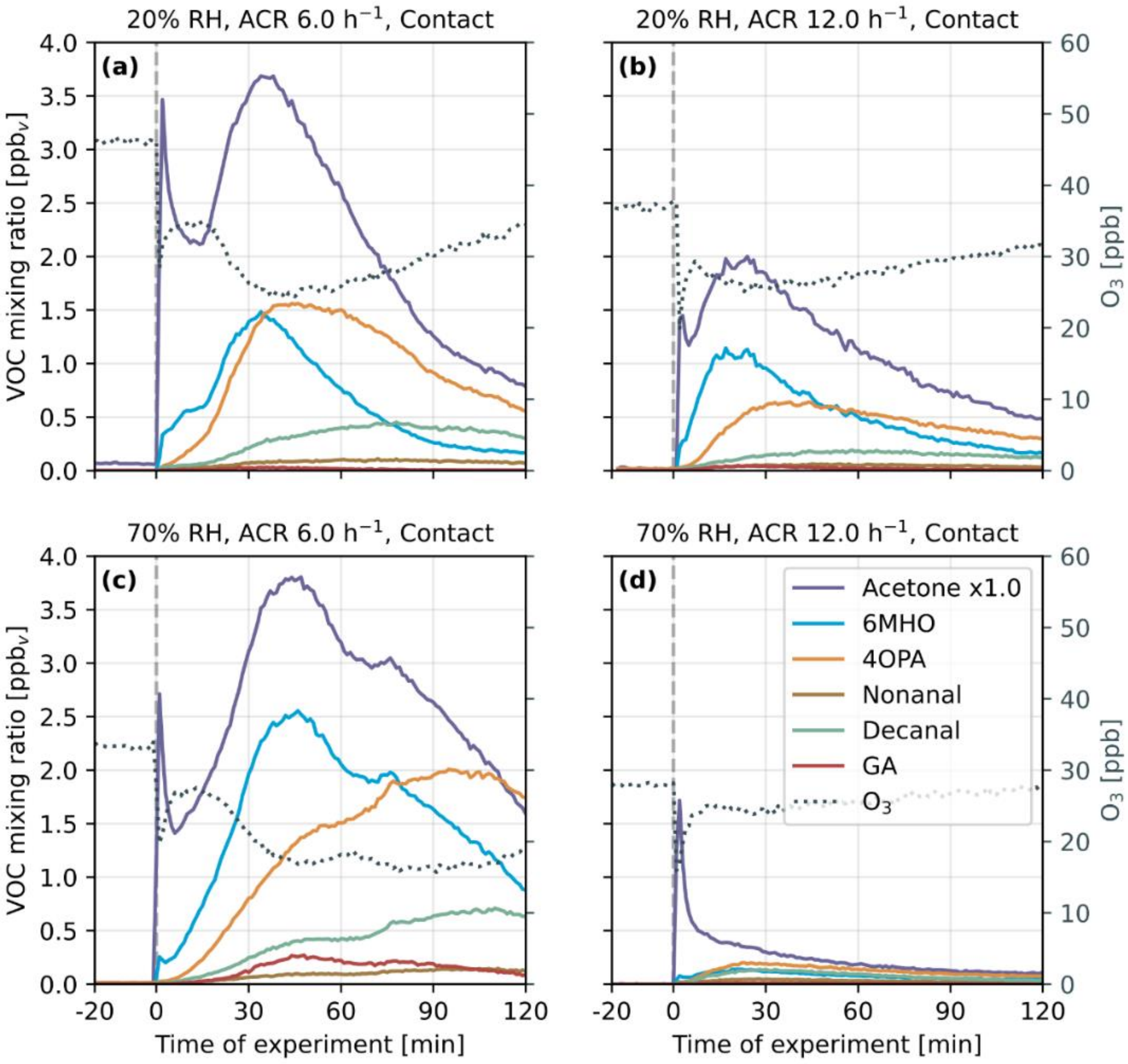

Figure S8. Mixing ratios of the monitored compounds for the duration of the experiments under different measurement conditions in the '**Dermal contact'** scenario and the corresponding O_3 mixing ratios. The signals include the 6MHO and acetone interference. The measurement shown in subplot (d) was likely affected by a leak in the experimental setup.

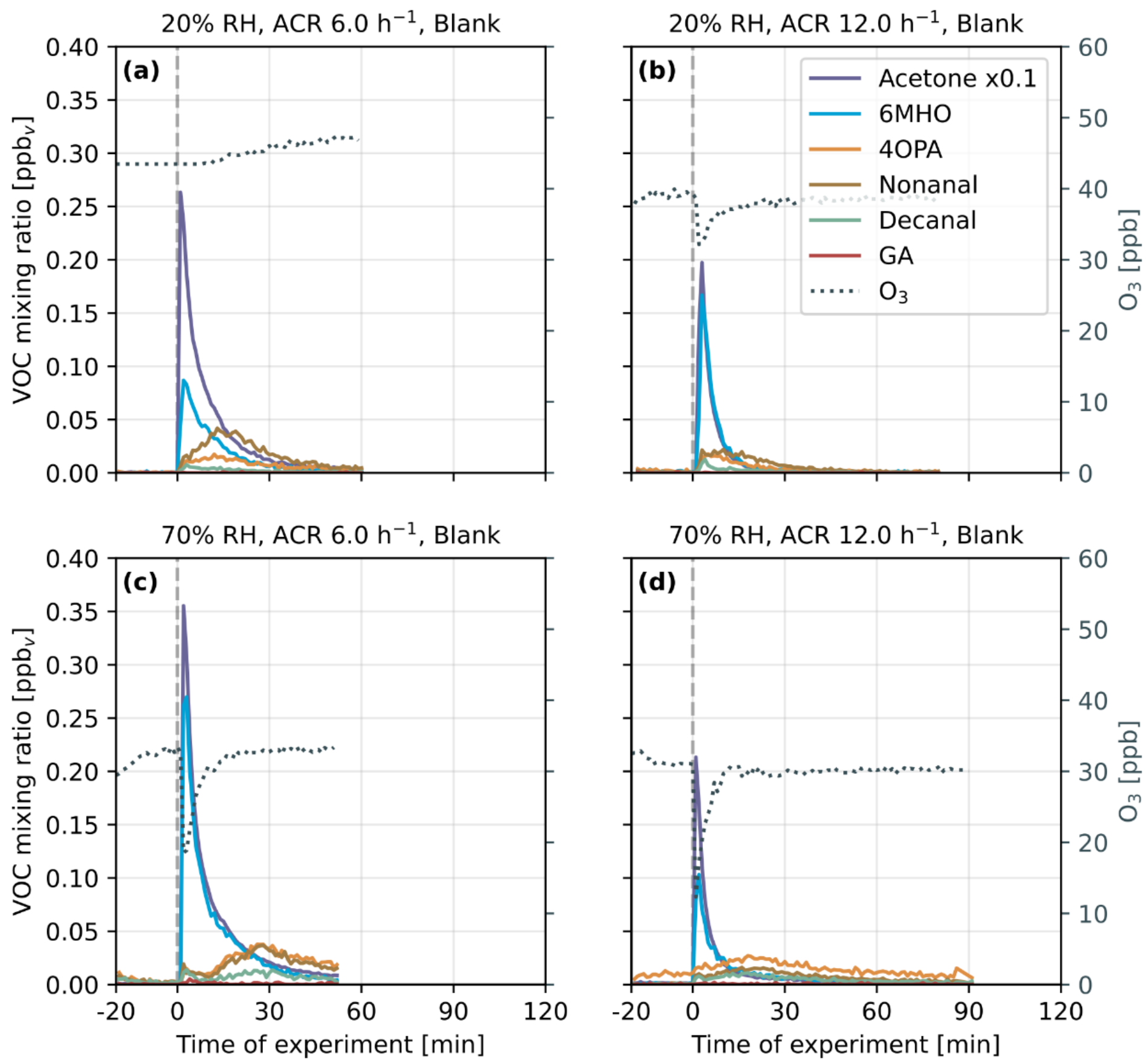

Figure S9. Blank measurements under different experimental conditions, with what are likely contributions from the ambient air. Note the scaling of the acetone mixing ratios for clarity.

Supporting Text S3. Signal interference correction

When the chamber (desiccator) was opened briefly to place the glass plates within, there was a short influx of ambient air containing a non-negligible amount of acetone and 6MHO (see the blank measurements in Figure S9). To account for the resulting interference, peaks defined by a linear increase and logarithmic decay were fitted to the blanks measured at different air flow rates and humidity levels, as shown in Figure S10a. These peaks were then re-fitted to the acetone and 6MHO tracers measured at the corresponding conditions and subtracted from them, producing the corrected tracers (see Figure S10b). Tracers without this correction are shown in Figures S5-S9.

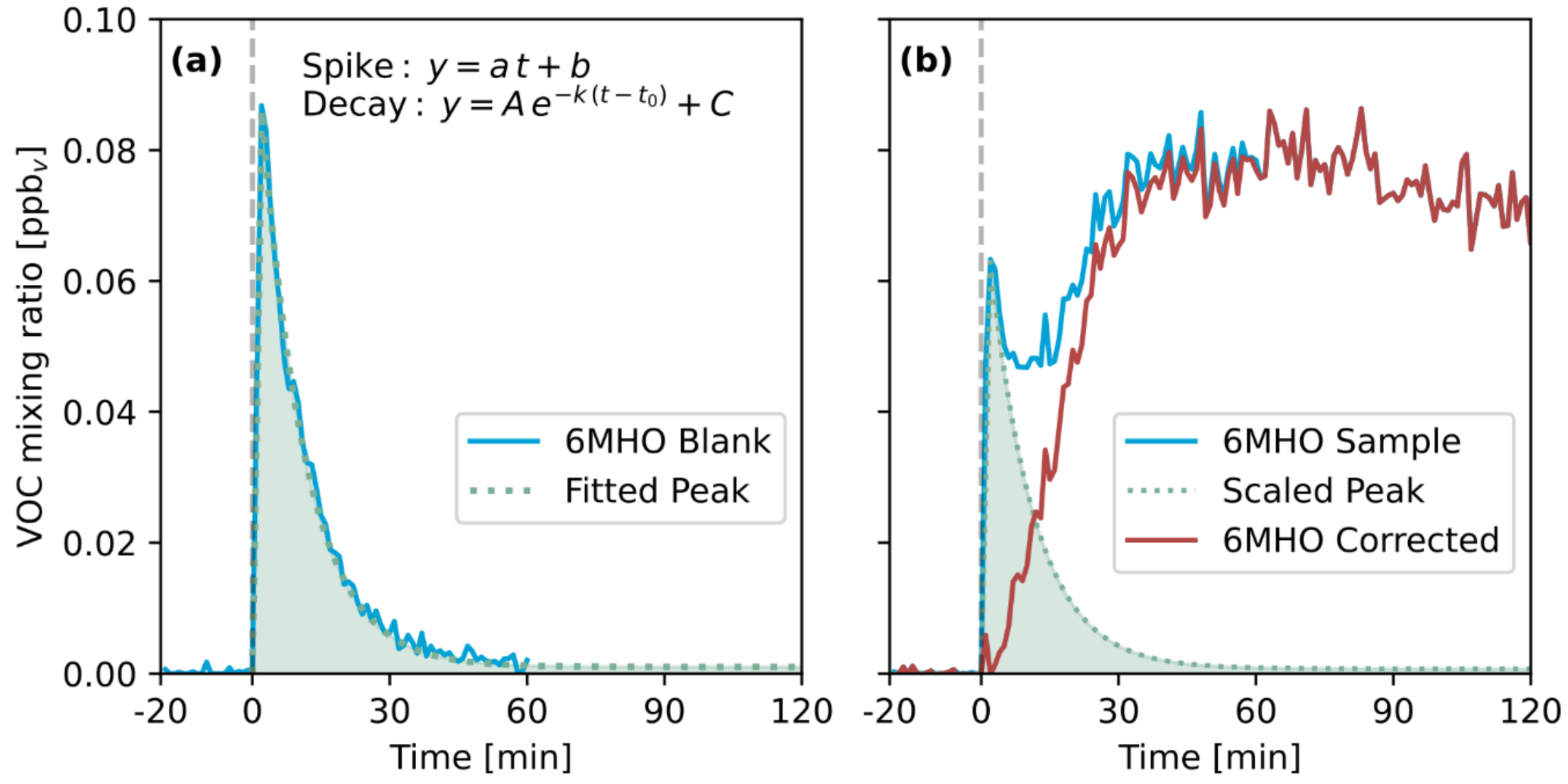

Figure S10. (a) Ambient air interference peak fitted to the blank measurement 6MHO tracer and **(b)** the peak re-fitted to the sample signal, together with the resulting corrected tracer.

Table S5. Reaction products in the gas phase from all experiments in nanomoles for the duration of the experiment.

Soiling condition	Scenario	Acetone	4OPA	6MHO	Geranyl acetone	Nonanal	Decanal
Blank	20% 6 h^{-1}	n.d.	0.04	n.d.	n.d.	0.09	0.01
Blank	20% 12h^{-1}	n.d.	0.05	n.d.	n.d.	0.09	0.03
Blank	70% 6 h^{-1}	n.d.	0.10	n.d.	<0.01	0.09	0.03
Blank	70% 12h^{-1}	n.d.	0.22	n.d.	n.d.	0.10	0.07
Vertical	no ozone	n.a.	n.a.	n.a.	n.a.	n.a.	n.a.
Vertical	20% 6 h^{-1}	0.34	0.06	n.d.	n.d.	0.03	0.01
Vertical	20% 12h^{-1}	0.20	0.06	n.d.	n.d.	0.02	0.01
Vertical	70% 6 h^{-1}	0.07	0.03	<0.01	n.d.	0.02	0.01
Vertical	70% 12h^{-1}	0.18	0.03	0.02	n.d.	0.02	0.01
Horizontal	no ozone	n.a.	n.a.	n.a.	n.a.	n.a.	n.a.
Horizontal	20% 6 h^{-1}	0.08	0.08	0.01	n.d.	0.20	0.02
Horizontal	20% 12h^{-1}	n.d.	n.d.	n.d.	n.d.	0.08	0.00
Horizontal	70% 6 h^{-1}	0.11	0.03	0.01	n.d.	0.13	0.03
Horizontal	70% 12h^{-1}	n.d.	0.02	n.d.	n.d.	0.05	0.01
Freshly soiled	no ozone	n.a.	n.a.	n.a.	n.a.	n.a.	n.a.
Freshly soiled	20% 6 h^{-1}	8.10	1.22	0.65	0.01	0.10	0.35
Freshly soiled	20% 12h^{-1}	5.02	0.99	0.87	0.04	0.68	0.33
Freshly soiled	70% 6 h^{-1}	9.65	3.60	3.08	0.16	0.34	1.42
Freshly soiled	70% 12h^{-1}	8.03	2.33	2.98	0.22	0.28	1.12
Dermal contact	no ozone	n.a.	n.a.	n.a.	n.a.	n.a.	n.a.
Dermal contact	20% 6 h^{-1}	20.3	10.3	6.27	0.13	0.84	3.32
Dermal contact	20% 12h^{-1}	23.5	9.51	10.2	0.38	1.04	3.36
Dermal contact	70% 6 h^{-1}	26.9	15.7	16.6	1.57	1.12	5.20
Dermal contact	70% 12h^{-1}	3.50	2.41	1.44	0.10	0.57	1.54

n.a = not analysed; n.d. = not detected

Table S6: Target analytes product amounts relative to blank-corrected ΔO_3, given in %.

		Gas phase						Surface					
		Acetone	4OPA	6MHO	GA	Nonanal	Decanal	6MHO	GA	C22 ald.	Σ gas phase	Σ surface	Σ both
Freshly	20% 6 h^{-1}	12.7	1.91	1.02	0.02	0.16	0.55	4.64	2.11	5.77	16.3	12.5	28.8
	20% 12h^{-1}	5.90	1.16	1.02	0.05	0.80	0.39	2.06	0.86	2.10	9.32	5.02	14.3
	70% 6 h^{-1}	10.3	3.85	3.29	0.17	0.36	1.52	3.33	1.24	3.11	19.5	7.68	27.2
	70% 12h^{-1}	11.5	3.35	4.28	0.32	0.40	1.61	3.13	1.52	5.10	21.5	9.75	31.2
Dermal	20% 6 h^{-1}	12.6	6.43	3.91	0.08	0.52	2.07	0.00	0.09	0.05	25.6	0.14	25.8
	20% 12h^{-1}	12.8	5.19	5.55	0.21	0.57	1.83	0.29	0.19	0.04	26.2	0.52	26.7
	70% 6 h^{-1}	19.2	11.2	11.8	1.12	0.80	3.70	0.50	0.09	0.06	47.8	0.65	48.4
	70% 12h^{-1}	22.3	15.3	9.17	0.64	3.63	9.80	0.00	0.70	0.51	60.9	1.21	62.1

Note: Surface Σ values in this table include measured surface 6MHO. In Figures 4 and 5, surface 6MHO is excluded from the plotted surface-yield totals for the reason discussed in the manuscript.

Table S7: Target analytes product amounts relative to Δsqualene, given in %.

		Gas phase						Surface					
		Acetone	4OPA	6MHO	GA	Nonanal	Decanal	6MHO	GA	C22 ald.	Σ gas phase	Σ surface	Σ both
Freshly	20% 6 h^{-1}	21.5	3.24	1.72	0.03	0.27	0.93	7.88	3.58	9.79	27.67	21.25	48.91
	20% 12h^{-1}	15.1	2.97	2.61	0.12	2.04	0.99	5.26	2.19	5.38	23.81	12.82	36.64
	70% 6 h^{-1}	24.0	8.96	7.66	0.40	0.85	3.53	7.74	2.89	7.24	45.40	17.86	63.26
	70% 12h^{-1}	28.7	8.32	10.6	0.79	1.00	4.00	7.79	3.79	12.7	53.43	24.25	77.68
Dermal	20% 6 h^{-1}	69.9	35.6	21.6	0.45	2.90	11.5	0.00	0.48	0.27	142.0	0.75	142.7
	20% 12h^{-1}	78.2	31.7	33.9	1.27	3.47	11.2	1.74	1.12	0.26	159.7	3.13	162.9
	70% 6 h^{-1}	86.9	50.6	53.5	5.06	3.61	16.8	2.27	0.42	0.26	216.4	2.95	219.4
	70% 12h^{-1}	10.9	7.53	4.50	0.31	1.78	4.81	0.00	0.34	0.25	29.9	0.59	30.47

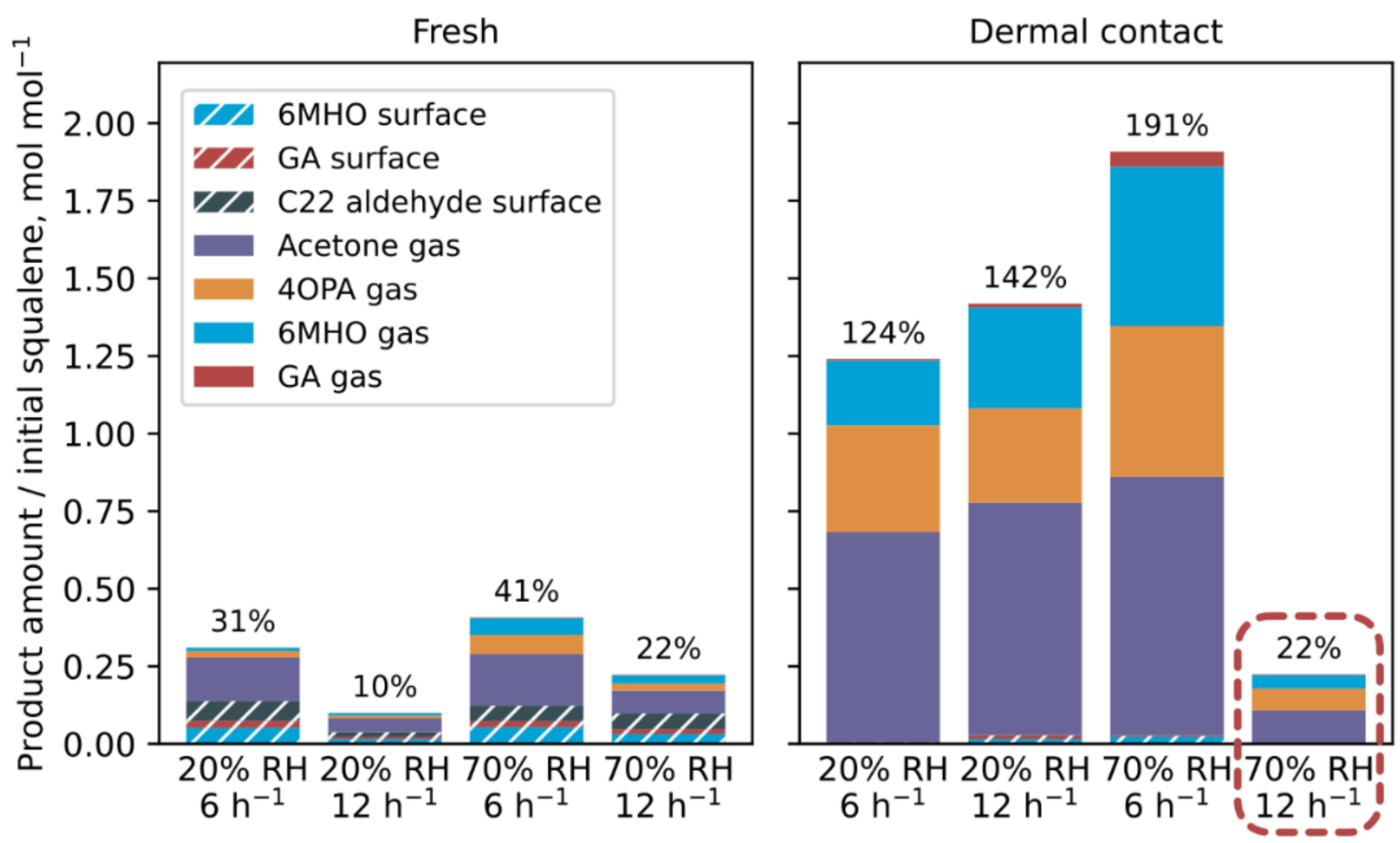

Figure S11: Gas- and surface-phase product amounts normalised to the initial amount of squalene for the Fresh and Dermal contact scenarios under different relative humidity and air-change-rate conditions. Note that the data shown for the Dermal contact 70% RH, 12 h^{-1} scenario are almost certainly affected by an experimental error.

Table S8. Reaction products on the surfaces from all experiments in nanomoles per surface (plate); some measured values below the LOD are given for reference. C_{22}-aldehyde = 4,9,13,17-Tetramethyl-4,8,12,16-octadecatetraenal (quantified in squalene equivalents).

Soiling condition	Scenario	6MHO	GA	Nonanal	Decanal	C_{22} aldehyde	C_{15}-acid
Blank	20% 6 h^{-1}	*0.18	*0.04	1.01	*0.37	<0.08	<6.2
Blank	20% 12h^{-1}	*0.28	*0.04	1.01	*0.34	<0.08	<6.2
Blank	70% 6 h^{-1}	*0.54	*0.06	0.73	*0.32	<0.08	<6.2
Blank	70% 12h^{-1}	*0.49	*0.03	0.76	*0.32	<0.08	<6.2
Vertical	no ozone	n.d.	n.d.	1.06	1.20	<0.08	<6.2
Vertical	20% 6 h^{-1}	n.d.	n.d.	1.07	1.06	<0.08	<6.2
Vertical	20% 12h^{-1}	n.d.	n.d.	1.77	0.84	<0.08	<6.2
Vertical	70% 6 h^{-1}	n.d.	n.d.	0.61	*0.36	<0.08	<6.2
Vertical	70% 12h^{-1}	n.d.	n.d.	0.83	0.91	<0.08	<6.2
Horizontal	no ozone	6.16	0.93	2.91	2.98	<0.08	17
Horizontal	20% 6 h^{-1}	1.74	*0.11	2.40	2.10	<0.08	19
Horizontal	20% 12h^{-1}	3.29	*0.10	2.30	1.29	<0.08	16
Horizontal	70% 6 h^{-1}	2.45	*0.13	1.98	1.20	<0.08	16
Horizontal	70% 12h^{-1}	0.80	*0.09	1.88	1.07	<0.08	13
Freshly soiled	no ozone	n.d.	0.34	5.63	4.7	<0.08	70
Freshly soiled	20% 6 h^{-1}	2.97	1.35	6.81	44.2	3.69	79
Freshly soiled	20% 12h^{-1}	1.75	0.73	12.41	11.1	1.79	104
Freshly soiled	70% 6 h^{-1}	3.11	1.16	8.24	42.7	2.91	79
Freshly soiled	70% 12h^{-1}	2.18	1.06	8.77	7.83	3.55	92
Dermal contact	no ozone	1.12	0.71	0.76	1.60	<0.08	11
Dermal contact	20% 6 h^{-1}	n.d.	*0.14	1.88	10.5	<0.08	13
Dermal contact	20% 12h^{-1}	*0.50	0.34	2.69	10.2	<0.08	14
Dermal contact	70% 6 h^{-1}	0.70	*0.13	1.73	5.90	<0.08	14
Dermal contact	70% 12h^{-1}	n.d.	*0.11	1.29	5.87	<0.08	15

*measured but below LOD; n.d. = not detected

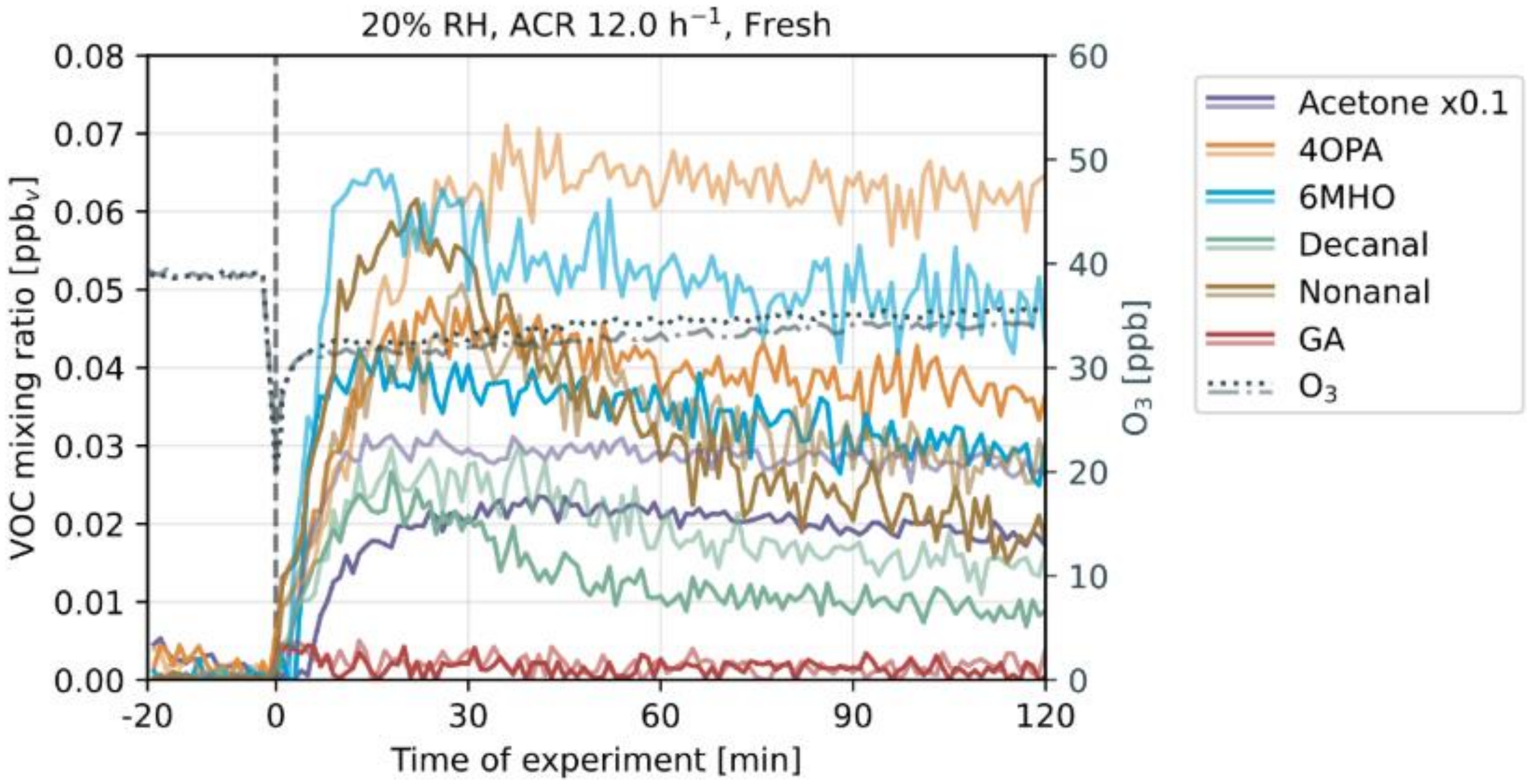

Figure S12. Corrected mixing ratios from a replicate measurement and the corresponding changes of O_3 mixing ratios measured at the outlet. Note the scaling of the acetone mixing ratio for clarity. The plot represents the duplicate experiment at 20% RH and ACR of 12 h^{-1} with the freshly soiled surface. The initial amounts of squalene were 95.6 and 129 nanomoles/plate, respectively, and ΔO_3 values were 107 and 118 nanomoles, respectively. The soiling occurred in a home environment, and the amount of freshly-shed squames on the plates was enhanced by shaking a slept-in t-shirt of a single subject over them.

Table S9. Results from the analysis of a wipe sample collected from the forehead of one team member.

Compound	µg in sample	% in sample
Squalene	130.9	77%
6MHO	0.23	0.1%
GA	0.45	0.3%
Nonanal	0.79	0.5%
Decanal	3.55	2.1%
C_{17} trienal	2.49	1.5%
C_{22} tetraenal	2.77	1.6%
C_{27} pentaenal	4.23	2.5%
C_{15}-acid	25.1	15%
